# Accessing Developmental Information of Fossil Hominin Teeth Using New Synchrotron Microtomography-Based Visualization Techniques of Dental Surfaces and Interfaces

**DOI:** 10.1371/journal.pone.0123019

**Published:** 2015-04-22

**Authors:** Adeline Le Cabec, Nancy Tang, Paul Tafforeau

**Affiliations:** 1 ESRF—The European Synchrotron, 71, avenue des Martyrs, CS 40220, F-38043 Grenoble, Cédex 9, France; 2 Department of Human Evolutionary Biology, Harvard University, Cambridge, MA, 02138, United States of America; 3 Department of Human Evolution, Max Planck Institute for Evolutionary Anthropology, Deutscher Platz 6, D-04103, Leipzig, Germany; 4 Department of Preventive Medicine, Exposure Biology Laboratory, Icahn School of Medicine at Mount Sinai, New York, New York, United States of America; Team 'Evo-Devo of Vertebrate Dentition', FRANCE

## Abstract

Quantification of dental long-period growth lines (Retzius lines in enamel and Andresen lines in dentine) and matching of stress patterns (internal accentuated lines and hypoplasias) are used in determining crown formation time and age at death in juvenile fossil hominins. They yield the chronology employed for inferences of life history. Synchrotron virtual histology has been demonstrated as a non-destructive alternative to conventional invasive approaches. Nevertheless, fossil teeth are sometimes poorly preserved or physically inaccessible, preventing observation of the external expression of incremental lines (perikymata and periradicular bands). Here we present a new approach combining synchrotron virtual histology and high quality three-dimensional rendering of dental surfaces and internal interfaces. We illustrate this approach with seventeen permanent fossil hominin teeth. The outer enamel surface and enamel-dentine junction (EDJ) were segmented by capturing the phase contrast fringes at the structural interfaces. Three-dimensional models were rendered with Phong’s algorithm, and a combination of directional colored lights to enhance surface topography and the pattern of subtle variations in tissue density. The process reveals perikymata and linear enamel hypoplasias on the entire crown surface, including unerupted teeth. Using this method, highly detailed stress patterns at the EDJ allow precise matching of teeth within an individual’s dentition when virtual histology is not sufficient. We highlight that taphonomical altered enamel can in particular cases yield artificial subdivisions of perikymata when imaged using X-ray microtomography with insufficient resolution. This may complicate assessments of developmental time, although this can be circumvented by a careful analysis of external and internal structures in parallel. We further present new crown formation times for two unerupted canines from South African Australopiths, which were found to form over a rather surprisingly long time (> 4.5 years). This approach provides tools for maximizing the recovery of developmental information in teeth, especially in the most difficult cases.

## Introduction

Scholars have long recognized the wealth of information preserved in dental hard tissues. Tooth microstructure has been used to study developmental defects, tooth formation times, and age at death (reviewed in [1–3]). Further, a strong correlation between dental development and important life history events has been suggested in human and non-human primates (reviewed in [4–7]). Life history can be described as series of developmental milestones in an individual’s life including birth, the duration of breast-feeding or weaning, as well as more complex aspects such as inter-birth intervals and lifespan; these events happen at different times and over different durations following species.

In order to determine growth periods and/or age at death of juveniles using a direct measurement independent of modern human or great ape standards, long-period incremental growth lines that course through the enamel (Retzius lines) and manifest on crown surfaces (perikymata) are the most commonly counted developmental features. Their periodicity however needs to be determined for calculating the crown formation time. This is achieved by counting the daily prism cross-striations between two successive long-period lines. This process is conventionally done by analyzing slices through the main cusp axis of the tooth under a microscope, and requires physical sectioning of the tooth. Other methods rely solely on the counts of cross-striations for determining age at death [8–10], but these approaches are extremely difficult to apply on fossils due to the high variability in the visibility of their enamel microstructure. In either case, these techniques are destructive, and are thus only rarely applied to valuable fossil specimens.

Retzius [11] was one of the first to observe long-period incremental growth lines in enamel on thin sections of vertebrate teeth, increments which have since been widely defined and described, especially in primates [12–16]. Their etiology is still poorly understood [15,17–21], although several explanatory hypotheses have been proposed to account for this growth disturbance: systemic origin [8,13,22], shift in the synchronization of different cellular biological rhythms [17] or even gastric disturbance (indigestion) caused by periodic feast days [23]. Structural variants of regular Retzius lines have been described, such as staircase-type Retzius lines [18] and S-shaped Retzius lines [24] while other lines running parallel to the developing front have been documented, e.g., irregular striae [19], pathological Wilson bands [25–27], and chevron lines [28]. When Retzius lines reach the outer enamel surface (OES), they manifest as continuous wave-like structures around the circumference of the crown [12–15,17,29]. First named perikymata by Preiswerk [30], these features are separated by grooves or imbrication lines following Pickerill’s description [31]. As early as 1854, Kölliker was one of the first to draw attention to the continuity between perikymata and Retzius lines which has then been confirmed by others [12,15,17,29–33]. Newman and Poole [17] and others [34,35], reviewed in [36–38] established the parallel between incremental growth features in enamel (Retzius lines, perikymata, cross-striations) and those existing in dentine (Andresen lines, periradicular bands, von Ebner lines). Dean [37] reports that periradicular bands are difficult to see because they are often packed close together, are shallower than perikymata, and lie beneath cementum, although they have been employed for reconstructions of developmental time [39]. Stress events often manifest as circumferential bands known as linear enamel hypoplasias on crown surfaces, in addition to irregular accentuated lines within the enamel (reviewed in [40–42]).

Determination of the timing of these defects may provide insight into stress, and may facilitate matching synchronously-developing teeth within a dentition (e.g., [41,43–46]). However, quantifying microscopic incremental features and documenting stress timing is often a serious challenge, since the clarity of Retzius lines within teeth is variable, and precise quantification of perikymata and hypoplasias is complicated by variation in their expression and the curvature of tooth surfaces [20,42]. Growth lines in teeth are conventionally observed from naturally-fractured teeth and histological (thin) sections under transmitted light microscopy. Nonetheless, perikymata counting from well-preserved tooth surfaces has the advantage of being non-destructive [47] and can be performed under a stereomicroscope or using scanning electron microscopy (SEM) with some preparation of the specimen (e.g., high resolution casts and sputter coating) [41], (reviewed in [48,49]]. Attempts for developing semi-automatic counting techniques of perikymata on isolated teeth with well-preserved outer enamel surfaces have so far demonstrated only limited success [50,51].

Histological assessments of incremental features often rely on physically-sectioned teeth, which limits material available for study, or on high-resolution impressions of tooth surfaces, which require accessible tooth germs or erupted teeth with well-preserved lateral surfaces. However, over the last decade, developments of propagation phase contrast X-ray synchrotron microtomography (PPC-SR-μCT) have permitted virtual histology, or non-destructive imaging of the internal aspects of dental tissues (e.g., [6,52–54]). In addition to being non-destructive, PPC-SR-μCT data may be used to produce high resolution 3D models of OES, as well as virtual section planes of various orientations and thicknesses, which improve the visibility of growth lines (e.g., [6]). Propagation phase contrast scans reveal the interface between two materials as a double fringe (adjacent black and white halves, with the white being on the side of the denser material), which yields sharper surfaces than absorption scans. Importantly, the real physical interface between two materials is at the exact junction between these white and black fringes.

In brief, materials are characterized by their index of refraction (n) which is a combination of attenuation (β or μ) and phase shifts (δ), as n = 1- δ + iβ. Pure absorption occurs when the distance ‘D’ between the scanned object and the detector equals zero. In the case where transverse coherence of the beam is sufficient, such as in third generation synchrotron sources, when D increases but remains in the near field of the Fresnel diffraction region, phase dominates over absorption and the phase shifts resulting from the different densities of the matter become visible in the so-called ‘edge-detection regime’ [55,56]. In conventional CT, D remains small enough so that the phase is generally not detectable, except for small objects imaged at a resolution close to 1 μm, with average energies typically lower than 20 keV (see Figure 2 and the corresponding text in [57], and Figure 1 in [58]). The fringes related to the phase shifts represent one of the main advantages of synchrotron virtual histology for studies of dental development, as the phase contrast sensitivity to small density differences is orders of magnitude stronger than that of absorption (e.g., [52,59]). This approach facilitates the non-destructive observation of incremental growth lines in teeth, and yields exceptional microscopic clarity of surfaces and interfaces due to the strong phase fringes associated with these structures.

Here we describe and validate a new application of 3D virtual histology that enhances the identification and quantification of long-period growth lines on the OES, and stress pattern on both the OES and the enamel-dentine junction (EDJ). This is an alternative approach to conventional methods to determine tooth crown formation times and developmental defects, especially in the case of teeth with altered surfaces. This 2D-3D approach has been used to determine the age at death in juvenile dentitions that cannot otherwise be studied. This is the case for unerupted teeth that are not observable with other techniques, specimens inaccessible using classical histology due to conservation issues or 2D synchrotron paleohistology due to poor preservation of internal structures. The techniques presented in this paper have been developed during a broad comparative study involving Plio-Pleistocene juvenile hominins [60,61] and of the MH1 *Au*. *sediba* holotype [62–64]. In the latter case, this combined 2D-3D approach has yielded age at death and overall dental development characterization despite poorly preserved external and internal structures. The goal of this paper is neither to challenge nor to solve potential methodological problems of previously published values of long-period line counts performed on teeth with good surface quality that are presently taken as references for comparison with our own results, but rather to propose new approaches to investigate specimens that would be inaccessible with other techniques.

The combination of 2D virtual histology and 3D high quality rendering of dental surfaces and interfaces facilitates detailed studies of fossil dentitions by enabling the use of any single fragmentary piece of information in a global approach. We present two case-studies of the lower canines of MLD2 (still enclosed in its crypt) and StW151, and we calculate their crown formation times. By maximizing the amount of information obtainable from rare and precious fossil specimens, this approach will allow us to better understand the evolution of human life history.

## Material and Methods

### Sample and phase contrast synchrotron μ-CT data acquisition

The sample under study comprises both *in situ* and isolated permanent developing and crown complete teeth of Plio-Pleistocene juvenile hominins. Details about specimens, sites, dating and tooth types are provided in S1 Table. In the following text, tables, and figures, teeth will be designated by abbreviations as follows: ‘L’ or ‘U’ for ‘lower’ or ‘upper’; ‘L’ or ‘R’ for ‘left’ or ‘right’; ‘I’, ‘C’, ‘M’ for ‘incisor’, ‘canine’ and ‘molar’; and finally the tooth position in Arabic numerals. For instance, ‘LRI2’ represents ‘lower right lateral incisor’.

The teeth were scanned using PPC-SR-μCT on the beamline ID 19 at the ESRF (Grenoble, France), with a voxel size of 4.96 μm and a propagation distance of four meters. Additional technical details are provided in S2 Table. Volumes were reconstructed using a filtered back-projection algorithm (PyHST2 software, ESRF). Original 32-bit stacks were converted into 16-bit tiff stacks. The subscans were then concatenated, ring artifact correction was applied on tiff slices when required, and the concatenated subscans were cropped so as to define the final size (bounding box) of the dataset. Finally, for general overviews, smaller volumes were generated using binning of 2*2*2, yielding datasets of about 10 μm pixel size, with improved signal to noise ratio. Depending on preservation quality and tooth size, either the full resolution data and/or the binning version were used for developmental analyses in 3D, but only the full resolution data were used for the virtual histological slices.

### 3D topo-densitometric rendering of the dental surfaces

Segmentation and 3D rendering were performed with VGStudio MAX 2.2 software (Volume Graphics Gmbh, Heidelberg, Germany, www.volumegraphics.com), and final image processing for light source combinations (detailed below) was made with Adobe Photoshop CS4 (v. 11.0.2) using automated script systems. The OES of each tooth was segmented by capturing the white fringe at the interface between air and enamel with region growing tools, yielding models of physical surfaces. The EDJ was typically captured by segmenting the black phase contrast fringe at the interface level, and then by adding the dentine itself into the segmentation mask.

**Fig 1:**
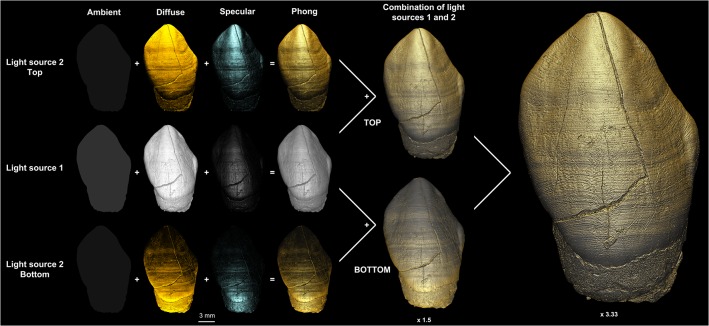
3D Phong rendering and colored light system. Principle of the 3D Phong rendering illustrated with the URC of MLD11-30. Illumination of the 3D model simultaneously by two light sources (LS1 and LS2), each composed of three components (three first columns): ambient, diffuse and specular. Each of the three rows (left half of the picture) shows the individual effect of each component. The combination of all light components is presented in the fourth column. LS1 (middle row) employs a white hue and is oriented in a perpendicular direction to the computer screen (viewer’s perspective—labial side of the crown). Light source 2 has a low white ambient (5 in VGStudio MAX 2.2), an orange diffuse light of moderate intensity (35) and a pale blue specular component with a tenfold higher intensity than the orange light (about 200). Combined with LS1, LS2 is oriented from the top (for taking a first set of images during the rotation of the tooth when mounting a multiple view plate, see S1 Fig), and then from the bottom (second set of images, same conditions) to light the 3D model with a low angle incidence to make topographical and densitometric details more visible. Both sets of images were then combined in Adobe Photoshop to enhance and sharpen topographic details with a mask of high frequency reinforcement. This operation involved taking the top-light image and subtracting structures smaller than 20 pixels that were also present in the bottom-light image (low frequencies), resulting in the combination of unique details from each direction in the final 3D model (far right). File name: Figure_1.tif.

The Phong 3D rendering available in VG Studio MAX 2.2 was chosen to optimize illumination of the 3D model. This algorithm takes into account the position of the observer and the specular properties (from the Latin *specularis*, ‘of a mirror’, i.e., relating to or having the qualities of a mirror) of the illuminated object [65,66]. The 3D model was lit simultaneously by two light sources (hereafter referred to as ‘LS1’ and ‘LS2’), each of which comprised three additive components of lights: (i) ambient (uniform intrinsic brightness of the surface independent of the direction); (ii) diffuse (light spread onto the whole surface, which reflects smooth portions of the surface); and (iii) specular (reflecting light from rough surfaces). Fig 1 illustrates how the orientations of the colored light sources are combined to light the 3D model and explains in detail the Photoshop combination protocol where the high frequencies from the set of top light pictures are superimposed to the bottom light set, and vice versa. Given the curved nature of tooth crown surfaces, this approach permits one to select a light orientation that best reveals features such as perikymata or hypoplasias (S1 Movie). Comparable light settings are also available in other 3D visualization software programs such as Avizo (XMeshPack).

**Fig 2:**
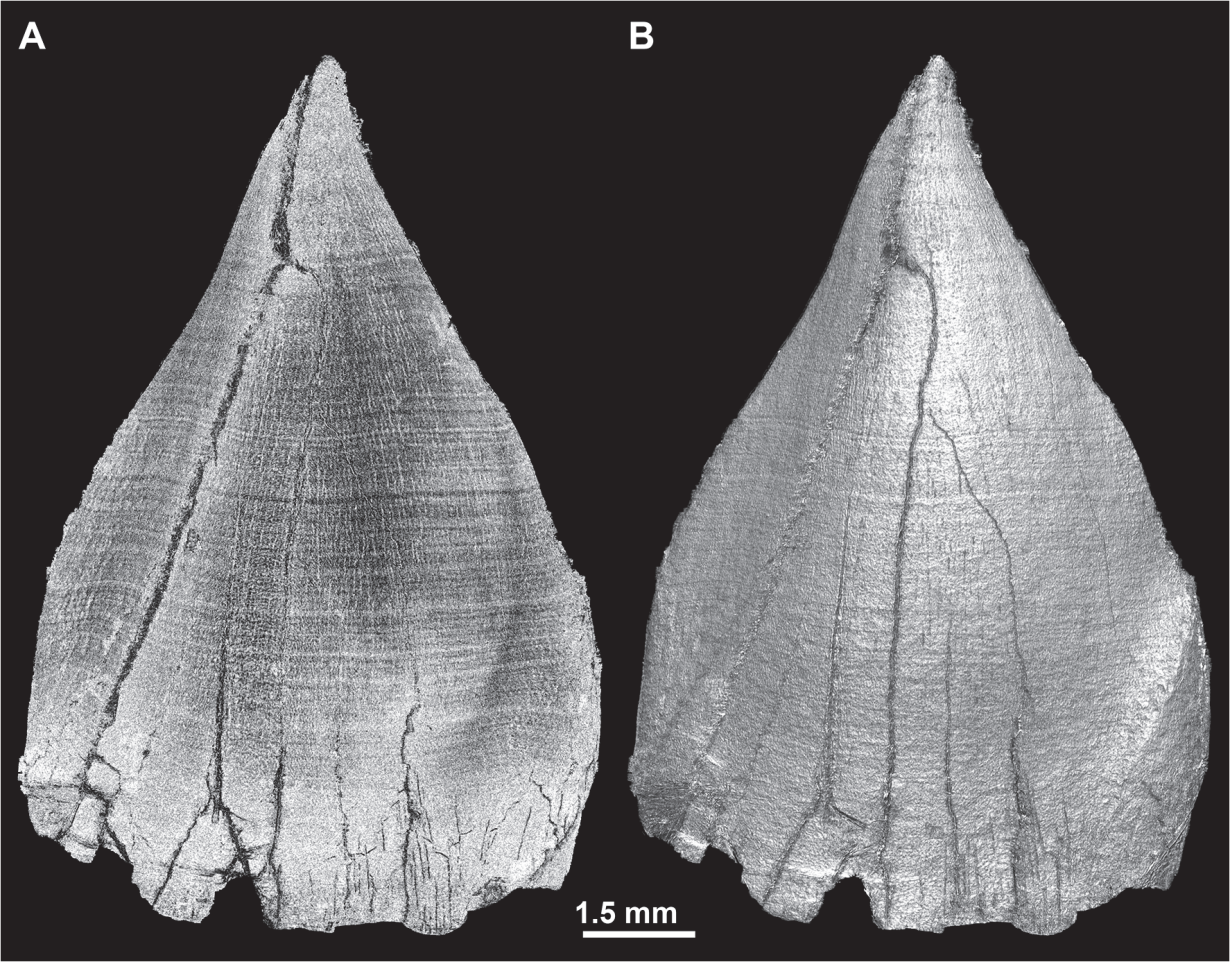
Topography and fine variation in density at the EDJ. Renderings of the EDJ of the ULC of StW151, with two white lights sources (default in VGStudio MAX 2.2). (A) The tooth rendered with ‘ScatterHQ’, which reveals only subtle density variation (gray values) at the EDJ. (B) The EDJ rendered with ‘Phong 3D’ and the ‘Normalize gradient’ commands; this renders only the topographical details of the EDJ surface, and omits shades related to density variation. File name: Figure_2.tif.

The same protocol was applied for visualizing the EDJ surface by segmenting the black fringe at the interface between enamel and dentine, and then by 3D Phong rendering using LS1 and LS2. As shown in Fig 2, two distinct types of information can be revealed on the EDJ: subtle variations of density and topographical relief. The OES density pattern is generally less informative than topography, hence the OES pictures are generated with the “normalize gradients” option in VGStudio MAX 2.2, which reveals topography only. Since this research deals both with 2D and 3D images of dental microstructure, we would like to underline that, although classical histology most commonly uses the term “accentuated lines” ([10], see [15,45] for a discussion about this issue), we will deliberately simplify by using “developmental defects” and “stress pattern”, which suits better for the description of both 2D and 3D data.

High resolution of the images produced and used for this paper will be made freely available online on the ESRF open-access database website at http://paleo.esrf.eu/.

To validate the accuracy and objectivity of this new approach for counting perikymata, we performed an inter-/intra-observer study as well as a comparison between stereomicroscope, SEM and our 3D renderings. All of these protocols and validation results are presented in S1 Supporting Information (Section I).

## Results

### High quality OES 3D renderings for perikymata counting

**Fig 3:**
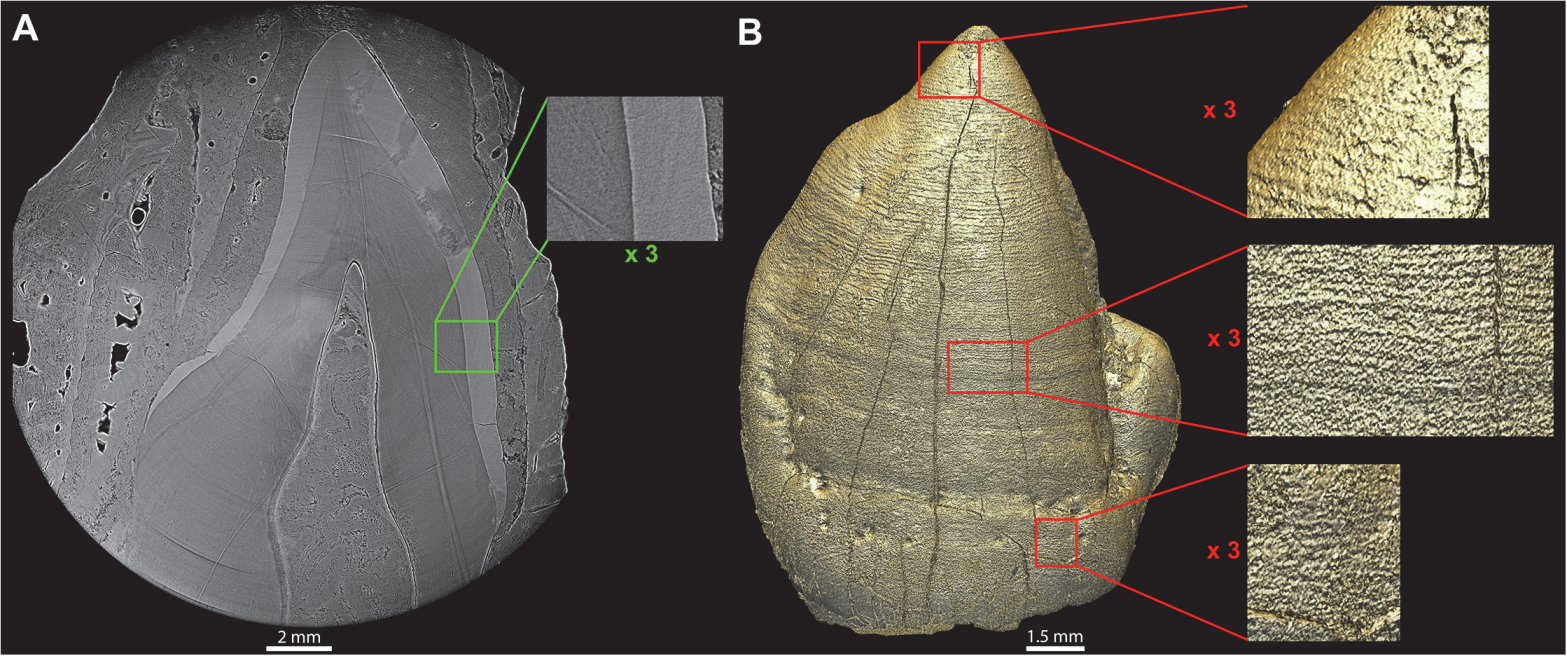
3D rendering of the unerupted LLC of MLD2 showing perikymata. The unerupted MLD 2 LLC in its alveolar crypt, which filled with matrix during fossilization. Retzius lines could not be revealed in the virtual histological data, in spite of changing thickness and orientation of the virtual 2D slice, the two thick lines are likely parts of ring artifacts (A). Despite continuous contact between the OES and the sediment filling the crypt, and the noisy nature of the fringes at the OES, the enamel surface could be successfully segmented and rendered (B), revealing countable perikymata almost all the way from the cusp tip to the cervix. Linear enamel hypoplasias are also apparent encircling the tooth crown. File name: Figure_3.tif.

**Fig 4:**
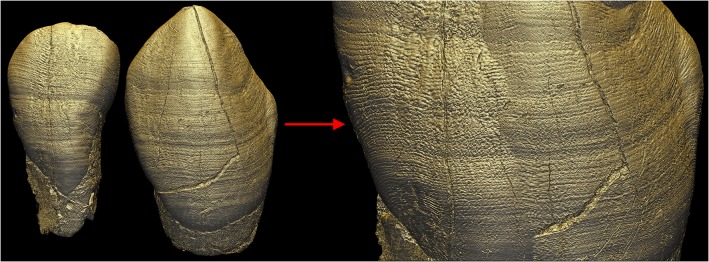
Enamel hypoplasia matching in the MLD11-30 URI2 and URC. Matching of the URI2 and URC of MLD11-30 based on linear enamel hypoplasias. On the left-hand side, both teeth are shown in natural proportions: the incisor (left) is smaller than the canine (right). The transformation on the far right was created by enlarging the incisor to be the same size as the canine, so that its hypoplasia and perikymata pattern matches the canine. S2 Movie shows the procedure for matching. File name: Figure_4.tif.

The combination of the sets of images obtained with LS2 oriented from the top and from the bottom improved the visibility of perikymata and facilitated counting from cervical to cuspal enamel (Fig 1 and S2 Fig). Perikymata near cusp tips could also be visualized for unworn teeth (S3 Fig). The bottom orientation of LS2 was the most optimized for visualizing the cervix, while the top orientation was best suited for the cusp tip and the cuspal half of the crown. A major advantage of this technique is the possibility to study well-preserved tooth germs that are still in the crypt, such as the LLC of MLD2 (Fig 3). For this tooth, perikymata can be reliably identified and counted from the cusp tip to the cervix, using different degrees of magnifications and areas of the labial aspect of the crown (see the results of the inter-/ intra-observer comparison in S1 Supporting Information, section I). Moreover, this technique permits the matching of hypoplasias across the dentition, as illustrated with the URI2 and URC of MLD11-30 (S2 Movie and Fig 4). In addition, and from a qualitative point of view, multiple light source orientations also reveal different sets of features related to attrition and wear (S3 Fig).

**Fig 5:**
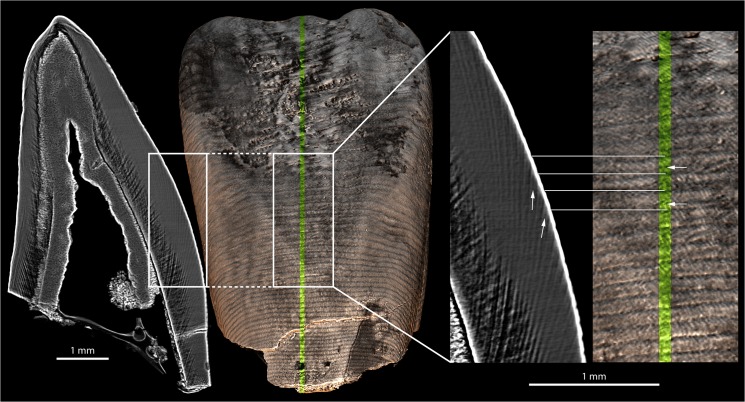
Retzius lines and perikymata doubling in the LLI1 of KB 5223. Virtual histological slices (grayscale images) showing subdivisions of Retzius lines (white arrows) in the LLI1 of KB5223 (labial view), and their corresponding expression as subdivisions of perikymata (white arrows) on the outer enamel surface. The dotted lines show the fidelity of 2D – 3D matching through horizontal alignment. The position of the labiolingual 2D section is indicated on the 3D model by the green stripe. See S1 Supporting Information (Section II) for a discussion about this phenomenon related to taphonomical alteration (local demineralization). File name: Figure_5.tif.

**Fig 6:**
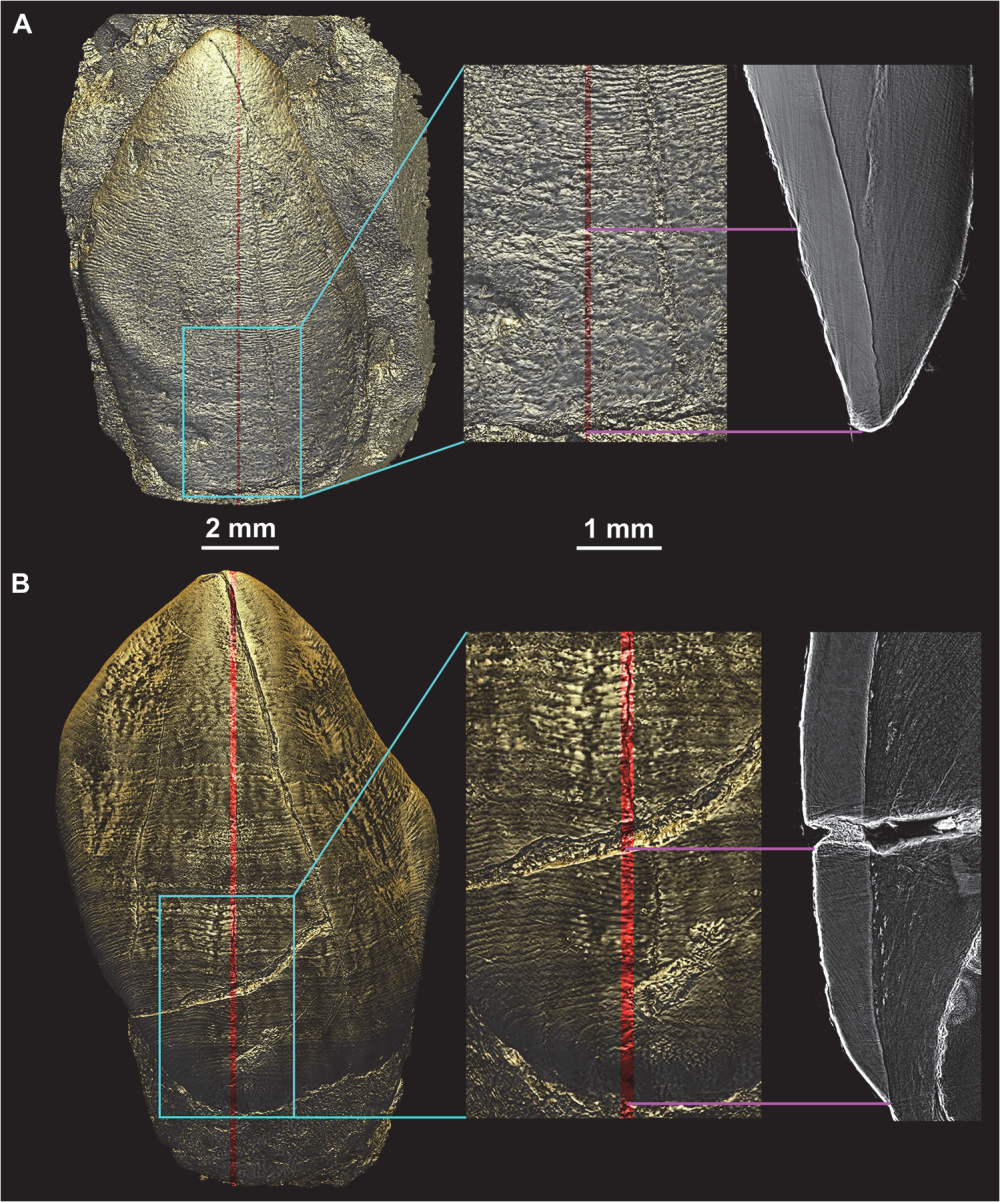
Complementarity of 2D and 3D developmental information illustrated for StW151 LLC and MLD11-30 URC. When calculating the crown formation time of StW151 LLC, we use Retzius lines on a 2D virtual slice in the cervical area, since the 3D model does not show clearly identifiable perikymata in that region (A). The cervix of the MLD11-30 LLC (B) yields the largest variation for both inter- and intra-observer counts (S2 Supporting Information, Tab “Average deciles”). This is due to the presence of unequal subdivisions of perikymata and a poor visibility of the perikymata at the very bottom of the cervix. The exact alignment between the 2D slice (its thickness explains that the alignment appears not exact with the 3D, although it is) and the 3D model of the OES is shown by pink lines at the cervix and at a hypoplasia for StW151, and at the bottom edge of a fracture for MLD11-30. File name: Figure_6.tif.

Fig 5 shows a clear correspondence between perikymata on 3D models and Retzius lines in 2D virtual slices. A similar relationship between hypoplasias on 3D models and accentuated lines in 2D slices is also illustrated in Fig 6.

In some regions, we note the existence of fine grooves between perikymata, which seem to correspond to internal subdivisions of Retzius lines (Fig 5) that could sometimes be misleading during long-period line counting. They occur locally in the middle of the traditionally defined structures, dividing Retzius bands, and sometimes perikymata into two roughly equivalent halves. We explore this phenomenon in S1 Supporting Information (Section II) and conclude that this is likely related to an increased visibility of these long-period line subdivisions caused by a local demineralization. This is particularly noticeable on the KB5223 teeth (see the virtual histological slices in [61] showing a clear degradation of the inner tissues of the KB5223 teeth). The internal subdivisions we observe seem similar to those described by FitzGerald [67], although their external expression as perikymata subdivisions are accentuated by X-ray imaging at too low resolution. The partial volume effect combined with the 5 μm pixel size of the PPC-SRμCT data induces these perikymata subdivisions that are in fact not real topographical structures on the surface when observed at higher resolution. This phenomenon has first been very confusing at the beginning of our observations and counting sessions. However, by investigating and understanding its causes, it appears that it can be distinguished from true perikymata by checking at the virtual 2D sections and at the continuity of the structure on the 3D of the outer enamel surface. Despite this, we therefore can rely on our results, but raise this important point to keep in mind when working with specimens strongly affected by taphonomical alterations. We propose that if this degradation is too pronounced, one should consider excluding the specimen from the sample study to avoid introducing bias.

### Determination of the LLC crown formation time of MLD2 and StW151

The unerupted MLD2 canine is crown complete, and over half of its root is already developed. The segmentation of its OES yielded a reasonably usable 3D model allowing for perikymata counts (Fig 3), despite being in direct contact with the mineral matrix. The StW151 canine has not yet achieved its lateral enamel formation, although based on the general morphology of the tooth germ, it appears close to crown completion. The perikymata cannot be counted in the cervical area due to poor preservation of the tooth surface in this area, and Retzius lines could not be counted in the cuspal part of the tooth. Thus we combined Retzius line counts on a 2D virtual section of the cervical half of the crown, and perikymata counts on the 3D model for the cuspal half (Fig 6). Because the cervix of the tooth has yet to form and an accurate projection of its completion is practically unattainable, our estimate represents a minimal crown formation time (Table 1). The perikymata counts used for the crown formation times of MLD2 and StW151 are the average of the three observers’ means (S3 Table and S2 Supporting Information). The long-period line periodicity is 8 days for StW151 and 7 days for MLD2, determined by high resolution synchrotron virtual histology [61]. The cuspal enamel thickness was measured on virtual 2D sections oriented in the developmental plane (passing through the dentine horn tip and the pulp chamber roof in the middle of the labial aspect). We attempted to directly measure the cuspal daily secretion rates (further abbreviated as CuDSR) on three hominin canines. Unfortunately, since high resolution data for cuspal enamel were not available, we based these measures on cuspal Retzius lines, observed on two orthogonal cut planes (S11 Fig and S1 Supporting Information, section III). This yielded an average CuDSR of 3.57 μm/d obtained on the maxillary canines of STW151, MLD11-30 and STS2. In addition, since this approach remains less precise than direct observations of cuspal cross-striations along prisms, we investigated the effect of using different CuDSR (following tooth type and taxon) found in the literature to provide a range of crown formation times (CFT) that maximizes the probability of containing the true value. The cuspal rates range from 3 μm/d in recent modern human canines to 6.06 μm/d in the molar of South African *Homo* DNH35. Those results are explained and reported in S1 Supporting Information and S3 Supporting Information. We estimate that the MLD2 canine crown formed in 5.0 (±0.25) years, while the nearly complete StW151 canine crown required more than 4.8 (±0.15) years (average following our measured CuDSR, see Table 1 for total ranges). We report a wider range of CFT taking into account CuDSR of *Pan* and recent human canines as: 4.86–5.16 (±0.25) years for the MLD2 LLC and 4.61–4.92 (±0.15) years for the StW151 LLC. The widest range of the CuDSR values we have collected would lower the minimal CFT down to 4.69 (±0.25) years for the MLD2 LLC and to 4.39 (±0.15) years for the StW151 LLC, using the CuDSR of the South African *Homo* DNH35 at 6.06 μm/d [61]. Although it is incomplete, the StW151 canine would have likely have formed in the same time or over slightly more time than the MLD2 canine.

**Table 1:** Determination of crown formation times for the mandibular left canines of MLD2 and of StW151.

	StW151			MLD2		
	*Minimum*	Maximum	Measured	Minimum	Maximum	Measured
Cuspal daily secretion	6.06	2.64	3.57	6.06	3.00	3.57
rate (CuDSR) [μm/day]						
Source	CuDSR DNH35	CuDSR *Homo*	Mean	CuDSR DNH35	CuDSR *Homo*	Mean
	South African	*sapiens*	measured rate	South African	*sapiens*	measured rate
	*Homo* molar [61]	canine [68]		*Homo* molar [61]	canine[68]	
Cuspal thickness [μm]		1285			1033	
Cuspal formation	212	404	370	171	345	297
time [days]						
Number of perikymata		174 (±7)			220 (±13)	
Long-period lines		8			7	
periodicity[61] [days]						
Lateral enamel formation		1392 (±56)			1540 (±91)	
time [days]						
Crown formation time	1604	1796	1762	1711	1885	1837
[days]		(±56)			(±91)	
Crown formation time	4.39	4.92	4.83	4.69	5.16	5.0
[years]		(±0.15)			(±0.25)	

The number of perikymata used for calculating the formation time of the lateral enamel is the average of the three observers' means. The true crown formation time of the StW151 LLC will exceed our results since the crown is not fully formed. The lateral enamel formation time was determined from the perikymata counts on the 3D renderings combined with 2D virtual histological slice (for StW151), multiplied by the long period lines periodicity. The cuspal enamel formation time was determined from a linear measurement from the dentine horn tip to the enamel surface at the level of the first perikymata, multiplied by enamel daily secretion rates (see S1 Supporting Information [section III] and S3 Supporting Information).

### Visualization and matching of the stress pattern on the EDJ

**Fig 7:**
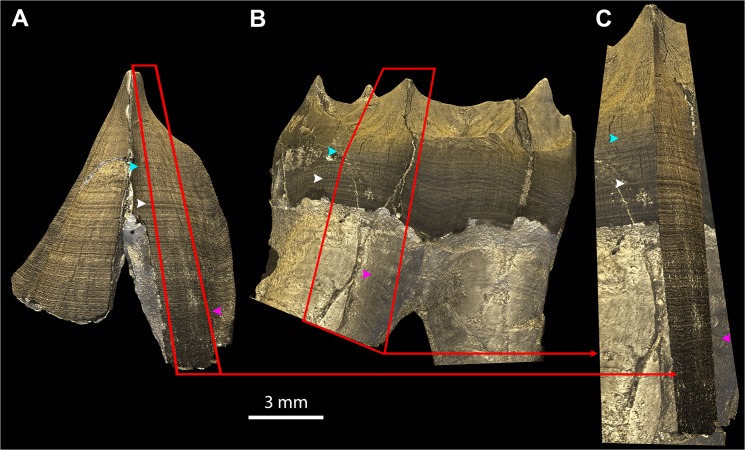
EDJ matching in multiple teeth of a single individual. The 3D models of the EDJs of the LRC (A) and LRM1 (B) of KNM-KP34725 are matched in (C) by superimposing a portion of each EDJ (red frames and arrows) following the stress pattern as a barcode on the EDJ and root surface (colored arrowheads). This is done with Abode Photoshop by rotation, translation, isometric scaling, perspective and skewing of the fragment of EDJ of the canine onto the fragment of molar that is taken as a reference. (The two apical thirds of the roots of the molar were out of the field of view during scanning, thus the roots appear to be cut in an abrupt manner.) File name: Figure_7.tif.

The colored light system and Phong 3D rendering reveal a stress pattern on the EDJ as alternating dark and light stripes of variable thickness and intensity, which may be thought of as a unique barcode. These stripes result from fine variations in density on either side of the EDJ. Given a similar thickness of incremental lines and defects in dentine and enamel, the smaller their angle of intercept with the EDJ, the broader the band will appear on the EDJ (S12 Fig). The variation in gray levels within the fringes themselves may yield this stress pattern on the 3D renderings of the EDJ (S13 Fig). The high sensitivity of this method results from the effect of superimposition of the stress pattern (and to a lesser extent the regular incremental pattern) with the phase contrast fringes at the EDJ level. This produces a very precise topography (e.g., well defined hypoplasias) with segmentation of the black fringe, and reveals subtle density variations due to stress pattern (Fig 2). The combination of these two aspects using the lighting system described above gives a novel 3D topo-densitometric rendering that reveals regular incremental features and irregular stress patterns (Fig 7). When an individual is represented by several developmentally overlapping teeth, the pattern on the EDJ can be matched across the teeth (S1 Table and Fig 7) following the same procedure described for perikymata and hypoplasia visualization (Fig 6 and S2 Movie).

## Discussion

### New techniques for long-period growth line visualization

High-quality propagation phase contrast synchrotron-based 3D renderings of tooth crown surfaces can be reoriented, rescaled, or illuminated to optimize the region of the tooth investigated (e.g., cervix versus cusp tip) and its topography (curved versus flat surface). The well-established correspondence between Retzius lines and perikymata [15,28,33,37,69,70] is illustrated in Fig 5, with an exact alignment of the 2D virtual slice and the 3D model of the OES. This approach aims for a more objective illustration and quantification of long-period lines and developmental defects due to the standardized and semi-automated protocol for light orientation and image generation. Our intra-/inter-observer pilot study shows that there is some variability in the long-period line identification due, in part, to the experience of observers (S1 Supporting Information, section I). Despite very different backgrounds and experiences in techniques for investigating dental development among the observers, our results generally yield reasonable agreement within and between observers, although some teeth showed greater variability. Explanations for this greater intra-/inter-observer variation may result from: 1) taphonomy/diagenesis of the tooth surfaces; 2) experience and ability of the observer to recognize a “real” structure, as opposed to the perikymata subdivisions observed in some cases on the PPC-SRμCT scans. In the case of a complex surface with hypoplasias and perikymata subdivisions, such as that of the MLD11-30 URC, the variability of counts was highest in deciles where subdivisions occur, as well as near the cervix. Our observations of perikymata and Retzius line subdivisions as a localized phenomenon (S7 Fig) draw the attention onto using X-ray microtomography on taphonomically altered enamel surfaces, as the partial volume effect on the OES can affect the data accuracy, in case of strong modification of the subsurface density (S1 Supporting Information, section II). We have demonstrated that the perikymata and Retzius lines subdivisions observed in the KB5223 incisors, and which run parallel to the real structures, are primarily related to authentic internal developmental features, but they are appearing on the 3D rendered surfaces due to a strong local demineralization of the enamel subsurface (see visible prisms on the enamel surface in the inset of S8 Fig-A; S1 Supporting Information, section II; S8 and S9 Figs). This can become a potential non-negligible pitfall for crown formation time calculation since it can lead to artificially inflated long-period line counts if not identified (also noted by [28]) and/or, in the most extreme cases, to an underestimation of the number of daily lines between long-period lines (periodicity). It however can be overcome by using a 2D-3D matching to check the relevance of the different structures. The presence of these additional lines may account for some of the differences between our counts and published values (S4 Fig) for the specimens the most affected, but the variability observed among the three raters remains in the range of the one in the published values. Additionally, we have shown on S6 Fig that a real structure visible as a thin layer partially covering the perikymata ridge can be imaged with binocular, SEM and PPC-SRμCT, proving that some of these perikymata subdivisions are not an imaging artifact related to X-ray techniques (S8 Fig-A, C and E). This is nevertheless still unclear whether or not these structures are a byproduct of the diagenetic modifications that affected the KB5223 specimen. This structure can yet not really be mistaken for the expression of a long-period line.

Although distinguishing between “real” and ancillary lines may sound straightforward since, in most of the cases it would result in unlikely periodicity values, this could become critical when the observer is faced with choosing between a periodicity of 6 or 12 days which are both possible values for hominins, or even 5 or 10 days (see reported ranges by [10,15,19,33,71–75]). We recommend to use a statistical approach for reporting a periodicity: in the high resolution scan, counts should cover several consecutive Retzius bands in several locations, so as to sample different conditions of preservation of the tissues in subsurface. In addition, we suggest that long-period lines values should be reported more generally as the average of multiple counts (which is certainly the case in previously published values, but is rarely written) and take the mode only if the results are congruent (S3 Table and S2 Supporting Information: second tab). In case of high uncertainty on the periodicity, crown formation times and age at death reports should reflect the range of long-period lines counts.

**Fig 8:**
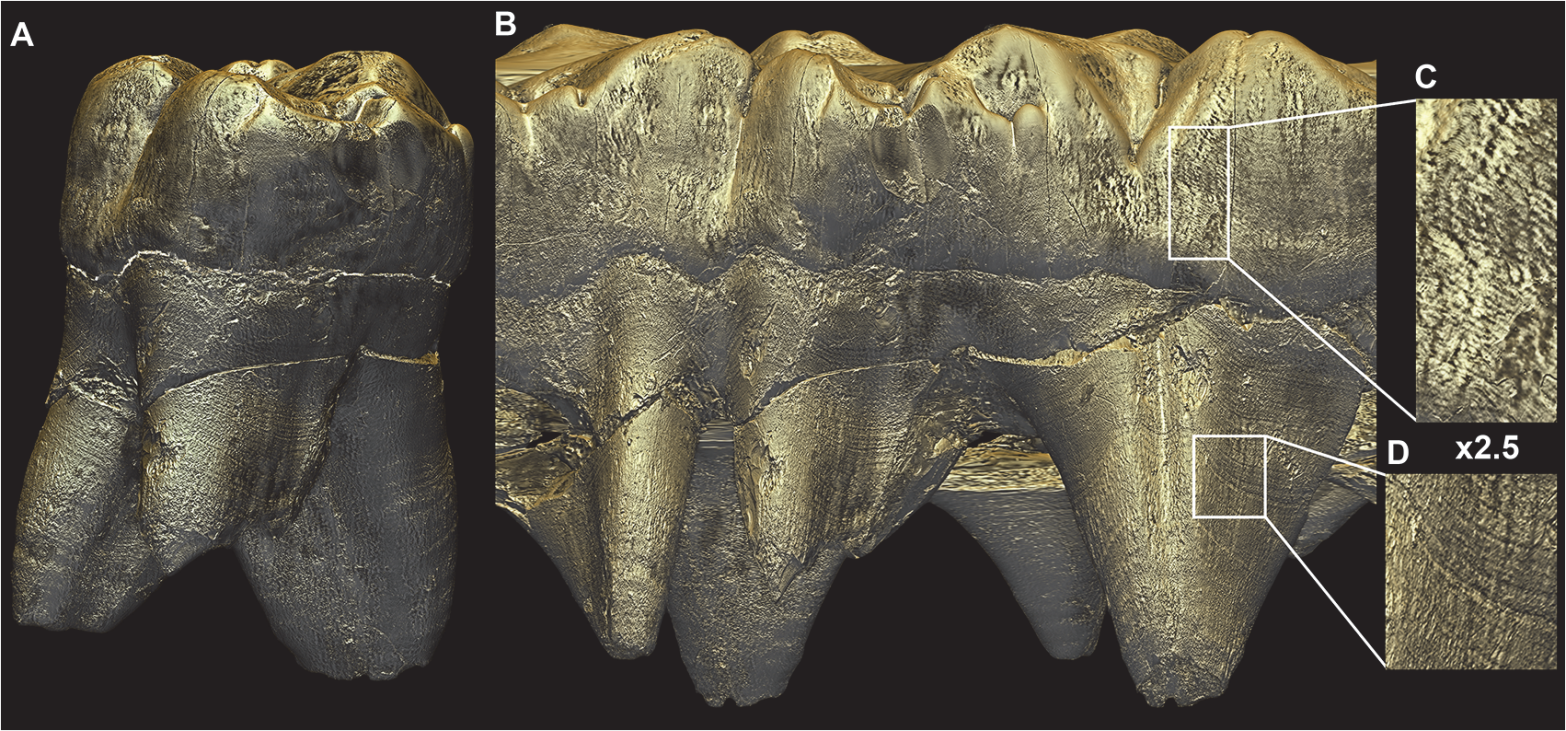
Unwrapped external surface of the StW151 ULM1. Virtual unwrapping of the outer surface of the StW151 ULM1 obtained from the concatenation of a single pixel-wide frames saved during the complete rotation of the tooth around its long axis. On the left side of the unwrapped surface, the tooth is viewed from the buccal side, and the mesio-buccal and disto-buccal roots can be seen in the front, while the lingual root is visible in the back. The lingual view, at the right of the unwrapped tooth, shows the lingual root in the front, and the two buccal roots in the back. Since not all points are at the same distance from the center of rotation of the tooth, some parts of the tooth can be distorted: the furcation area of the buccal roots is stretched in the middle of the unrolled tooth. Perikymata, periradicular bands and hypoplasias are visible and can be tracked across the tooth. Images are not to scale. File name: Figure_8.tif.

This new visualization approach is highly sensitive to subtle developmental disturbances, and is particularly useful for matching teeth when hypoplasias or accentuated lines are not visible on virtual 2D slices. Moreover, the same approach may be applied to visualize root surfaces (S1 Fig and Fig 8), which also show regular long-period lines (periradicular bands) and a stress pattern. A related approach consists of literally unwrapping the tooth (Fig 8): perikymata, hypoplasias, and periradicular bands can be followed around the circumference of the tooth. This information can then be used for stress matching across the dentition, or checking the consistency of a structure (imaging artifact vs. defect or perikymata subdivisions). This approach yields a comprehensive view of all the stress pattern and developmental aspects of a tooth in a single image, which is particularly illustrative for multi-cusped teeth.

The main drawback of synchrotron imaging is that samples are irradiated with a powerful X-ray beam, leading to the delivery of a high X-ray dose. This can lead to darkening of the enamel ([53], but see [76]) and may also have detrimental effects to potentially preserved ancient DNA (aDNA) [76]. The effects of synchrotron scanning on aDNA are currently being investigated, and preliminary results show that, nowadays, the configurations used for full sample imaging do not result in significant damage to aDNA [77]. In most of the cases, there is no darkening of the enamel. For the rare cases where this occurs, the natural color can be fully recovered after a few hours under low-power low-energy UV irradiation using “dark light” (main wavelength at 370 nm, close to visible light, *contra* [76]).

### MLD2 and StW151: surprisingly long-forming canines

Synchrotron imaging has the considerable advantage here of yielding access to unerupted *in situ* teeth. Crown formation time of both MLD2 and StW151 appear to be relatively long (> 4.5 years, Table 1) compared to values published for other specimens (e.g., [48]). Our values fall at the lower end of the range reported for female great apes in [68]. The perikymata count could be comparable to that observed on the ULC of the ARA-VP-6/1 holotype of *Ardipithecus ramidus* that is 193 perikymata yielding a crown formation time of 4.29 or 4.82 years following the estimation of its periodicity at 7 or 8 days [78]. We would like to underline here the high variability induced by the use of periodicity ranges in the final results. This parameter has indeed been shown to be highly variable even within one single taxon [61]. The direct determination of long-period line periodicity represents a major advantage of developmental studies performed using PPC-SRμCT [6,46]. Therefore, crown formation times should be considered extremely carefully when no direct determination of periodicity is available. Moggi-Cecchi et al. [79] report a shorter crown formation time for the StW151 LLC. We suspect that their perikymata counts in the cervical area have been underestimated, as we realized that Retzius lines were much easier to identify on the 2D virtual slice than perikymata on the cervical area of the OES of this tooth (S4 Supporting Information). Nonetheless, other instances of canine crowns developing over an even longer period of time have been documented for Plio-Pleistocene South African specimens [61]. Since these crowns were not accessible or were too damaged for direct observation (both external and internal structures), these specimens could not be fully quantified from virtual 2D slices. The approach applied in the current study demonstrates how developmental information may be retrieved from unerupted teeth, even in the case of poor preservation, by combining multiple observational techniques of PPC-SRμCT. The crown formation time of the MLD2 LLC is strikingly long, and could be interpreted as resulting from errors in the perikymata counts, because of the complex surface topography of this tooth. Nevertheless, the fact that the StW151 LLC presents a crown formation time at least as long as that of MLD2, and that multiple counts of the MLD2 canine by three different observers end within a limited variability of results comparable to that of other well-preserved teeth, confirms our initial conclusion about the MLD2 LLC. Our results demonstrate that canine crown formation time in South African Australopithecines and maybe early *Homo* (depending on the taxonomic attribution of StW151) can sometimes be far longer and more variable than expected from previously published studies. More extensive study is necessary to assess whether such long canine crown formation times may be related to taxonomical status, sexual dimorphism, or natural variability [70,68]. In future dental developmental studies involving PPC-SRμCT, not only should individual periodicity be directly determined as in previous studies [6,46] but also a special focus should be set on determining cuspal daily secretion rates in at least one anterior and one postcanine tooth. This would constraint the reported range and take into account taxonomic and anatomical (tooth class) variability.

### Stress pattern and its 3D visualization on the EDJ interface

**Fig 9:**
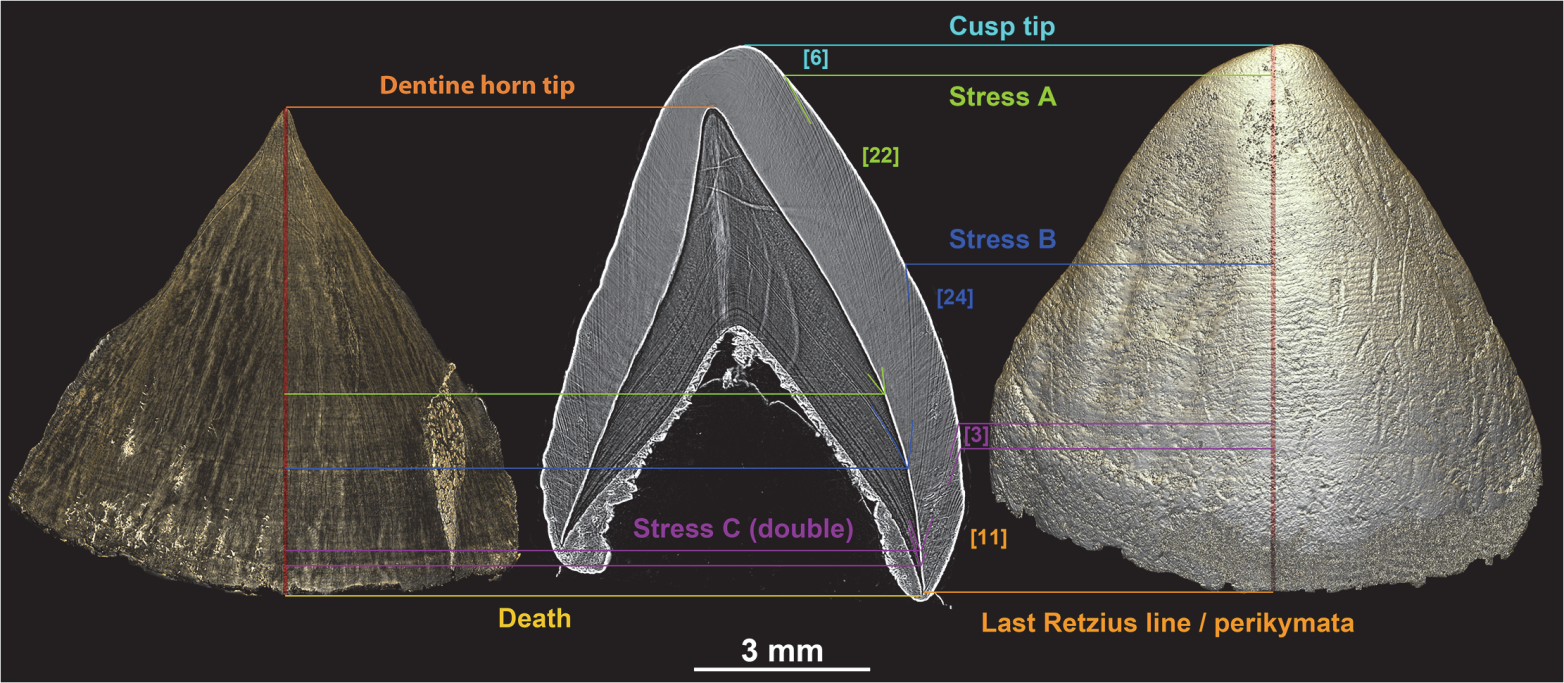
Direct correspondence of 2D and 3D developmental information. Matching of the incremental pattern between the standardized developmental slice of the ULC of STS2 and the 3D models of its EDJ and OES. Retzius lines or accentuated lines in the enamel on the 2D virtual slice allows the matching of a stress on the EDJ and OES. The number of long-period lines is indicated in square brackets between the major stress events highlighted on the 2D and 3D models, providing a quantitative overview of the time elapsed between stress. The long-period line count was performed on a high resolution image of the 2D slice to ensure a high definition of the growth lines. Further developmental information for this individual may be found in [61]. File name: Figure_9.tif.

Stress in enamel and dentine are commonly used to match teeth across a dentition as synchronous events [14,80–83]. Although odontoblasts secrete dentine slightly in advance of ameloblasts secreting enamel, this difference in time can be treated as negligible for general dental development studies [84]. For the first time, we reveal the stress pattern on the EDJ resulting from subtle variations of density and topography on both sides of this interface. This is possible because phase contrast reveals this information with high sensitivity in the black and white fringes at the interface between the two materials (S13 Fig). Although matching the EDJ (Fig 7) of several teeth does not yield temporal information, as the Andresen lines are rarely visible on the EDJ interface, it creates a relative chronology of stress events that then allows one to exploit any single usable piece of developmental information (periodicity, and number of perikymata, Retzius lines in enamel, Andresen lines in dentine) within that framework (Fig 9).

## Conclusion

The main advantages of the 2D-3D rendering protocol presented in this study are: (i) enhanced topographic and densitometric details of the OES and stress patterns on the EDJ; (ii) enhanced visibility of developmental structures from high-quality images allowing for reasonably consistent inter- and intra-observer agreement; (iii) accurate visualization of long-period growth increments in enamel and developmental defects using a combination of virtual histological 2D slices and 3D models; (iv) novel possibilities for visualizing surfaces of well-preserved teeth still in crypt; and (v) facilitation of the matching of stress patterns across an individual's dentition.

This 2D-3D combination rendering approach for visualization of dental surfaces opens up new possibilities for detailed developmental studies on exceptional fossil hominins with well-preserved partially complete developing dentitions. We however draw the attention on the impact that resolution and partial volume effect can have on demineralized areas in the enamel subsurface, when subdivisions of perikymata are observed on the 3D renderings. On the one hand, special care has to be taken to ensure reaching robust results based on perikymata counts derived from X-ray images. In the vast majority of cases, there is no possible mistake about recognizing real perikymata on PPC-SRμCT data. Further, it allowed for the first time to determine crown formation times of two unerupted early hominin teeth that would not have been fully accessible with any other technique. These results suggest that the short formation time conventionally expected for early hominin lower canine crowns may be too restrictive; the two specimens presented here showed development times of more than 4.5 years. Dental development in general should consider using direct determination of the individual’s periodicity to avoid reporting very wide ranges. In addition, future PPC-SRμCT-based developmental studies may improve by trying to measure systematically CuDSR, which would contribute to constrain even more the reported ranges for both crown formation times and age at death. This innovative approach is being employed to generate a comprehensive and permanent digital record of developmental information in invaluable and fragile fossil hominin specimens. These developmental data will be made freely available online and will thus facilitate future comparative studies.

## Supporting Information

S1 Fig Eight-view plate of the LLM1 of MLD2. The LLM1 of MLD2 imaged every 45° along the transverse axis following rendering by the protocol detailed here. This allows visualization of developmental defects or long-period features around the tooth to ascertain their authenticity (and rule out imaging or reconstruction artifacts). File name: S1_Fig.tif.
(TIF)

S2 Fig Rendered LLM1 of MLD2 showing the advantages of the combination of colored lights. Segmented enamel surface of the MLD2 LLM1 rendered in 3D using Phong’s algorithm. First, the model is lit with the two default light sources (the first is oriented perpendicular to the screen and coming towards the user, while the second is coming from the top) with all components having a white hue (A). Then, the second light source is successively oriented from the top (B) and bottom (C) with a low white ambient component, a moderate orange diffuse component, and a pale blue specular light. Two masks of reinforcement are then successively computed in Photoshop from the high frequencies of the top view (B) and the bottom view (C). These masks enhance the high frequencies of the views lit from the opposite direction: i.e., the mask computed from (B) enhances the high frequencies of (C) resulting in (E). The same is true for the mask computed from (C) reinforcing (B) and resulting in (D). Microscopic increments on the outer enamel surface (perikymata) are shown in corresponding insets of each lighting condition. File name: S2_Fig.tif.
(TIF)

S3 Fig Occlusal surfaces of the LLM1 (top row) and LLM2 (bottom row) of MLD2, lit with a combination of top and bottom colored second light sources. The LLM1 shows occlusal wear facets, macro- (A-C) and microwear (D) features. The unworn occlusal morphology of the LLM2 displays perikymata from the cusp tip downwards along the lateral enamel (E-G). File name: S3_Fig.tif.
(TIF)

S4 Fig Intra-observer variability in perikymata counts and comparison with published values. Whisker plot showing the variability in the perikymata counts for each observer, and the overall variability among observers, compared to perikymata counts published in the literature. File name: S4_Fig.tif.
(TIF)

S5 Fig Cumulative perikymata counts per decile reveals inter-observer variability. Cumulative plot of perikymata counts of all observers, per decile of crown height, for four teeth (STS24 URI1, MLD11-30 URC, SK62 LLI1 and the unerupted MLD2 LLC). ‘dec#1’ designates the first decile at the cusp tip, down to ‘dec#10’ at the cervix. File name: S5_Fig.tif.
(TIF)

S6 Fig Comparison of stereomicroscope, scanning electron microscope and synchrotron μCT approaches for perikymata visualization. Comparison of imaging techniques for perikymata visualization on the labial surface of the LRI2 of KB5223, with (A) stereomicroscope, (B) SEM, and (C) 3D rendering using PPC-SRμCT data (Phong, colored lights and normalize gradient in VGStudio MAX 2.2). File name: S6_Fig.tif.
(TIF)

S7 Fig Primary observation of perikymata subdivisions as a localized phenomenon. Three-dimensional model of the LLI2 of KB5223 (light source 2 is oriented from the top) showing subdivisions of perikymata (right inset). This phenomenon is only local, since there are no subdivisions for a contemporaneous area of the crown surface (top left inset). It is also not consistent through time, because there are no subdivisions in the cervical part of the crown (bottom left inset). S8 and S9 Figs reveal that this phenomenon can be induced by demineralization of the enamel sub-surface. See S1 Supporting Information (section II) for a discussion. File name: S7_Fig.tif.
(TIF)

S8 Fig Perikymata/Retzius lines subdivisions related to local demineralization of the enamel subsurface. Impact of tomographic partial volume effect on demineralized enamel sub-surface in the labial aspect of the KB5223 LLI2. The same region of interest is imaged at high resolution (0.7 μm voxel size, top row, A and B), 5 μm (middle row, C and D) and degraded 0.7 μm (bottom row, E and F) in an area showing perikymata/Retzius line subdivisions of equal size (green frame) on the 3D models (C and E) and the corresponding 2D virtual slices (location shown as a blue line on the 3D): 100 μm-thick (right) and inset showing the 5 μm-thick slice (left from the yellow frame). The turquoise inset illustrates the thin sheet of enamel overlaying the perikymata ridges, and which are not an artifact as it is also visible using other imaging techniques (see S6 Fig). Green arrows materialize the location the “true” long-period lines, while red arrows show the location of the subdivisions (not existing on A). File name: S8_Fig.tif.
(TIF)

S9 Fig Effect of PPC-CRμCT resolution on an area lacking demineralized long-period line subdivisions on the KB5223 LLI2. Imaging of a portion of the labial aspect of the KB5223 LLI2 crown not showing demineralized long-period line subdivisions, corresponding to same time period than the area presented in S8 Fig. Same configuration and color-coding as for S8 Fig (save for the turquoise inset that is not shown). It shows that the perikymata subdivisions presented in S8 Fig are induced by the partial volume effect when the resolution is not high enough to fully discriminate the external surface and the sub-surface demineralization. File name: S9_Fig.tif.
(TIF)

S10 Fig Evidence of non-artifactual perikymata subdivisions imaged by binocular microscope and PPC-SRμCT on the KB5223 LLI2. Perikymata subdivisions non-related to imaging artifacts are visible on the PPC-SRμCT-based 3D model of the KB5223 LLI2 (A, LS2 oriented from the bottom) and on the area framed in turquoise (in [A]) on the labial aspect of a silicone cast of the same tooth, observed under a binocular microscope (B). This illustrates the case where the demineralization of the enamel subsurface reaches the surface and creates a visible structure on the outer enamel surface. Green arrows point to true anatomical perikymata and red arrowheads show the subdivisions. The binocular picture is a courtesy of Dr. Tanya M. Smith. File name: S10_Fig.tif.
(TIF)

S11 Fig Methodology for direct cuspal daily secretion rate measurement on the STS2 ULC. The red cross illustrates where the measurement has been taken on the 3D model of the STS2 ULC (A), on 200 μm-thick sagittal (B) and transversal (C) slices in the cuspal enamel. Colored arrows show corresponding accentuated lines on both the transversal slice (C and E) and on magnified zone (D and F) from of the sagittal slice. Retzius lines are easier to follow as concentric lines on the transversal slices (C and E), and their tracing can be matched back onto the sagittal slice (B) for measuring cuspal distances to calculate the CuDSR. It has to be noted that C, D, E and F were enhanced with an unsharp filter in Adobe Photoshop CS4. File name: S11_Fig.tif.
(TIF)

S12 Fig Angle of intercept of stress lines at the EDJ. Illustration showing the importance of the angle of intercept of the long-period growth lines and developmental defects at the EDJ. A stress reaching the EDJ with an acute angle (here 30°/2) will manifest as a thicker band (3.7 A.U.; ‘A.U’ stands for ‘arbitrary unit’) than in the case of a larger angle (here 132°/2 corresponding to a thickness of 1 A.U. on the EDJ). A stress occurring early in dental development, such as the neonatal line in the permanent first molar, will be very strongly tangent to the EDJ because of the high extension rate during early cuspal enamel and dentine formation. This will result in a large band on the dentine horn tip. File name: S12_Fig.tif.
(TIF)

S13 Fig Stress on the EDJ results from density variation in the phase contrast fringes. Virtual 5 μm slice of the STS2 ULC through the PPC-SR-μCT dataset reconstructed in edge detection, where phase contrast reveals incremental long-period growth lines and a stress pattern (A). An inset shows the incremental growth lines in enamel and dentine, and developmental defects meeting at the EDJ (B). In the 3D plots shown in (B-E), both the surface relief (peaks) and coloring (“Spectrum” Look Up Table often used for topographical coding in Geographical Information System) represent the intensity of the gray values in both the black and white fringes, similar to topographic elevation. The higher the peak, the brighter the gray value will be in the white fringe (up to dark blue) or the darker the gray value in the black fringe (up to red). For the same portion of the tooth, for the white (C) and the black (D) fringes are shown as isolated. A detail of the white (above) and black (below) fringes at the EDJ (E) showing the peaks for the gray values levels where stress lines, generally of lower densities, reach the EDJ (color-coded arrows also shown in B-D; red for the black fringe and blue for the white fringe). These peaks in gray levels yield stress patterns that may be read as a “barcode” on the segmented 3D dentine models, and are visualized as bright or dark stripes of various width using Phong 3D rendering and colored light sources. File name: S13_Fig.tif.
(TIF)

S1 Movie Application of the Phong 3D rendering and of the colored light sources. The MLD2 LLM1 is lit with the first light source that is white and oriented to the front (not shown). Colors (for the diffuse and specular components) are given to the second light source (shown on the movie), which changes its orientation progressively to reveal different details of the enamel surface following the angle of incidence. The combination of LS2 oriented from the top and from the bottom is displayed after, showing a much sharper enamel surface with clear perikymata and stress. File name: S1_Movie.avi.
(AVI)

S2 Movie Enamel hypoplasia matching in the MLD11-30 URI2 and URC. MLD11-30 URI2 and URC matching based on linear enamel hypoplasia. Both teeth are first shown in their normal proportions, followed by a transformation that involves increasing the size of the incisor so that its stress pattern matches the one of the canine. In this example, the precision allows merging the two surfaces and corresponding perikymata. File name: S2_Movie.avi.
(AVI)

S1 Supporting Information Perikymata Counting: Intra- / Inter-Observer Agreement Study and Comparison between Imaging Techniques; Perikymata subdivisions; Cuspal daily secretion rates. File name: S1_Supporting_Information.docx.
(DOCX)

S2 Supporting Information Perikymata Counting: original data, decile distributions and descriptive statistics. File name: S2_Supporting_Information.xlsx.
(XLSX)

S3 Supporting Information Measured and published cuspal daily secretion rates for enamel cuspal formation of StW151 and MLD2 LLC. File name: S3_Supporting_Information.xlsx.
(XLSX)

S4 Supporting Information Comparison of perikymata count per mm of crown height between Moggi-Cecchi *et al*. (1998) [79] and this study for the StW151 LLC. File name: S4_Supporting_Information.xlsx.
(XLSX)

S1 Table Sample of fossil hominin teeth included in this study. File name: S1_Table.docx.
(DOCX)

S2 Table Scanning parameters for the acquisition of phase contrast synchrotron micro-CT data. File name: S2_Table.docx.
(DOCX)

S3 Table Inter-observer agreement study for all counting sessions (mean ± standard deviation). File name: S3_Table.docx.
(DOCX)

## References

[1] Smith TM. Incremental dental development: Methods and applications in hominoid evolutionary studies. J Hum Evol. 2008;54: 205–224. 18045649 10.1016/j.jhevol.2007.09.020

[2] Dean MC. Retrieving chronological age from dental remains of early fossil hominins to reconstruct human growth in the past. Philos Trans R Soc B Biol Sci. 2010;365: 3397–3410. 10.1098/rstb.2010.0052 20855313 PMC2981956

[3] Schwartz GT. Growth, Development, and Life History throughout the Evolution of *Homo* . Curr Anthropol. 2012;53: S395–S408.

[4] Smith HB, Crummett TL, Brandt KL. Ages of eruption of primate teeth: A compendium for aging individuals and comparing life histories. Am J Phys Anthropol. 1994;37: 177–231. 10.1002/ajpa.1330370608

[5] Dean CM. Tooth microstructure tracks the pace of human life-history evolution. Proc R Soc B Biol Sci. 2006;273: 2799–2808. 10.1098/rspb.2006.3583 17015331 PMC1664636

[6] Smith TM, Tafforeau P, Reid DJ, Pouech J, Lazzari V, Zermeno JP, et al Dental evidence for ontogenetic differences between modern humans and Neanderthals. Proc Natl Acad Sci U S A. 2010;107: 20923–20928. 10.1073/pnas.1010906107 21078988 PMC3000267

[7] Dean MC, Elamin F. Parturition lines in modern human wisdom tooth roots: do they exist, can they be characterized and are they useful for retrospective determination of age at first reproduction and/or inter-birth intervals? Ann Hum Biol. 2014;41: 358–367. 10.3109/03014460.2014.923047 24932749

[8] Boyde A. Estimation of age at death of young human skeletal remains from incremental lines in the dental enamel. Third International Meeting in Forensic Immunology, Medicine, Pathology and Toxicology. London; 1963. pp. 36–46.

[9] Antoine D. Evaluating the periodicity of incremental structures in dental enamel as a means of studying growth in children from past human populations University College London 2000.

[10] Antoine D, Hillson S, Dean MC. The developmental clock of dental enamel: a test for the periodicity of prism cross‐striations in modern humans and an evaluation of the most likely sources of error in histological studies of this kind. J Anat. 2009;214: 45–55. 10.1111/j.1469-7580.2008.01010.x 19166472 PMC2667916

[11] Retzius A. Bemerkungen über den innern Bau der Zähne, mit besonderer Rücksicht auf den im Zahnknochen vorkommenden Röhrenbau. Arch Anat Physiol Wiss Med. 1837; 486–566.

[12] Pantke H. Untersuchengen uber Retzius-streifen und Perikymatien. Stoma (Lisb). 1957;10: 32–40.

[13] Dean MC. The developing dentition and tooth structure in hominoids. Folia Primatol (Basel). 1989;53: 160–176. 2606394 10.1159/000156414

[14] Boyde A. Developmental interpretations of dental microstructure. Primate Life Hist Evol Monogr Primatol. 1990;14: 229–267.

[15] Risnes S. Growth tracks in dental enamel. J Hum Evol. 1998;35: 331–350. 9774498 10.1006/jhev.1998.0229

[16] Ramirez Rozzi F. Can enamel microstructure be used to establish the presence of different species of Plio-Pleistocene hominids from Omo, Ethiopia? J Hum Evol. 1998;35: 543–576. 9774510

[17] Newman HN, Poole DFG. Observations with scanning and transmission electron microscopy on the structure of human surface enamel. Arch Oral Biol. 1974;19: 1135–1143. 4531875 10.1016/0003-9969(74)90242-8

[18] Risnes S. Structural characteristics of staircase-type Retzius lines in human dental enamel analyzed by scanning electron microscopy. Anat Rec. 1990;226: 135–146. 2301733 10.1002/ar.1092260203

[19] Dean MC, Scandrett AE. The relation between long-period incremental markings in dentine and daily cross-striations in enamel in human teeth. Arch Oral Biol. 1996;41: 233–241. 8735009 10.1016/0003-9969(95)00137-9

[20] Guatelli-Steinberg D. What can developmental defects of enamel reveal about physiological stress in nonhuman primates? Evol Anthropol Issues News Rev. 2001;10: 138–151. 10.1002/evan.1027

[21] Hogg RT. Dental microstructure and growth in the Cebid Primates. PhD Dissertation, City University of New York. 2010.

[22] Asper H. Über die “Braune Retzius’sche Parallelstreifung” im Schmelz der menschlichen Zähne. Schweiz Vierteljahrsschr Für Zahnheilkd. 1916;26: 275–314.

[23] Gysi A. Metabolism in adult enamel. Dent Dig. 1931;37: 661–668.

[24] Dean MC, Shellis RP. Observations on stria morphology in the lateral enamel of *Pongo*, *Hylobates* and *Proconsul* teeth. J Hum Evol. 1998;35: 401–410. 9774502 10.1006/jhev.1998.0243

[25] Rose JC. Defective enamel histology of prehistoric teeth from Illinois. Am J Phys Anthropol. 1977;46: 439–446. 10.1002/ajpa.1330460309 871151

[26] Rose JC, Armelagos GJ, Lallo JW. Histological enamel indicator of childhood stress in prehistoric skeletal samples. Am J Phys Anthropol. 1978;49: 511–516. 10.1002/ajpa.1330490411 367176

[27] Rose JC. Morphological variations of enamel prisms within abnormal striae of Retzius. Hum Biol. 1979; 139–151. 457084

[28] FitzGerald CM. Do enamel microstructures have regular time dependency? Conclusions from the literature and a large-scale study. J Hum Evol. 1998;35: 371–386. 9774500 10.1006/jhev.1998.0232

[29] Risnes S. Rationale for consistency in the use of enamel surface terms: perikymata and imbrications. Eur J Oral Sci. 1984;92: 1–5. 10.1111/j.1600-0722.1984.tb00852.x 6585907

[30] Preiswerk G. Beitrage zur Kenntnis der Schmelzstruktur bei Saugetieren mit besonderer Beriicksichtigung der Ungulaten. PhD Dissertation, Universität Basel. 1895.

[31] Pickerill HP. The structure of enamel. Dent Cosm. 1913;55: 969–988.

[32] Kölliker A. Mikroskopische Anatomie oder Gewebelehre des Menschen. Engelmann; 1854.

[33] Dean MC, Beynon AD, Thackeray JF, Macho GA. Histological reconstruction of dental development and age at death of a juvenile *Paranthropus robustus* specimen, SK 63, from Swartkrans, South Africa. Am J Phys Anthropol. 1993;91: 401–419. 10.1002/ajpa.1330910402 8372933

[34] Kawasaki K, Tanaka S, Ishikawa T. On the daily incremental lines in human dentine. Arch Oral Biol. 1979;24: 939–943. 10.1016/0003-9969(79)90221-8 232978

[35] Newman HN, Poole DF. Dental enamel growth. J R Soc Med. 1993;86: 61 8369022 PMC1293839

[36] Dean MC. The nature and periodicity of incremental lines in primate dentine and their relationship to periradicular bands in OH 16 (*Homo habilis*). In: Moggi-Cecchi J, editor. Aspects of Dental Biology: Palaeontology, Anthropology and Evolution. Florence; 1995. pp. 239–265.

[37] Dean MC. Hominoid tooth growth; using incremental lines in dentine as markers of growth in modern human and fossil primate teeth In: Hoppa R, FitzGerald C, editors. Human Growth in the past Studies from bones and teeth. Cambridge University Press Cambridge; 1999 pp. 111–127.

[38] Smith TM, Toussaint M, Reid DJ, Olejniczak AJ, Hublin J-J. Rapid dental development in a Middle Paleolithic Belgian Neanderthal. Proc Natl Acad Sci. 2007;104: 20220–20225. 10.1073/pnas.0707051104 18077342 PMC2154412

[39] Smith TM, Reid DJ. Temporal nature of periradicular bands (‘Striae periradicales’) on mammalian tooth roots. Front Oral Biol. 2009;13: 86–92. 10.1159/000242397 19828976

[40] Goodman AH, Rose JC. Assessment of systemic physiological perturbations from dental enamel hypoplasias and associated histological structures. Am J Phys Anthropol. 1990;33: 59–110.

[41] Hillson S, Bond S. Relationship of enamel hypoplasia to the pattern of tooth crown growth: a discussion. Am J Phys Anthropol. 1997;104: 89–103. 9331455 10.1002/(SICI)1096-8644(199709)104:1<89::AID-AJPA6>3.0.CO;2-8

[42] Skinner MF, Pruetz JD. Reconstruction of periodicity of repetitive linear enamel hypoplasia from perikymata counts on imbricational enamel among dry-adapted chimpanzees (*Pan troglodytes verus*) from Fongoli, Senegal. Am J Phys Anthropol. 2012;149: 468–482. 10.1002/ajpa.22145 23041791

[43] Beynon AD, Dean MC, Reid DJ. Histological study on the chronology of the developing dentition in gorilla and orangutan. Am J Phys Anthropol. 1991;86: 189–203. 10.1002/ajpa.1330860208

[44] Dirks W, Reid DJ, Jolly CJ, Phillips‐Conroy JE, Brett FL. Out of the mouths of baboons: stress, life history, and dental development in the Awash National Park hybrid zone, Ethiopia. Am J Phys Anthropol. 2002;118: 239–252. 12115280 10.1002/ajpa.10089

[45] Schwartz GT, Reid DJ, Dean MC, Zihlman AL. A faithful record of stressful life events recorded in the dental developmental record of a juvenile gorilla. Int J Primatol. 2006;27: 1201–1219.

[46] Smith TM, Tafforeau P, Reid DJ, Grün R, Eggins S, Boutakiout M, et al Earliest evidence of modern human life history in North African early *Homo sapiens* . Proc Natl Acad Sci. 2007;104: 6128–6133. 10.1073/pnas.0700747104 17372199 PMC1828706

[47] Dean MC, Stringer CB, Bromage TG. Age at death of the Neanderthal child from Devil’s Tower, Gibraltar and the implications for studies of general growth and development in Neanderthals. Am J Phys Anthropol. 1986;70: 301–309. 3752228 10.1002/ajpa.1330700305

[48] Dean C, Leakey MG, Reid D, Schrenk F, Schwartz GT, Stringer C, et al Growth processes in teeth distinguish modern humans from *Homo erectus* and earlier hominins. Nature. 2001;414: 628–631. 10.1038/414628a 11740557

[49] Smith TM, Tafforeau P. New visions of dental tissue research: tooth development, chemistry, and structure. Evol Anthropol Issues News Rev. 2008;17: 213–226.

[50] Elhechmi I, Braga J, Dasgupta G, Gharbi T. Accelerated measurement of perikymata by an optical instrument. Biomed Opt Express. 2013;4: 2124–2137. 10.1364/BOE.4.002124 24156069 PMC3799671

[51] Bocaege E, Humphrey LT, Hillson S. Technical note: A new three-dimensional technique for high resolution quantitative recording of perikymata. Am J Phys Anthropol. 2010;141: 498–503. 10.1002/ajpa.21233 19953528

[52] Tafforeau P, Boistel R, Boller E, Bravin A, Brunet M, Chaimanee Y, et al Applications of X-ray synchrotron microtomography for non-destructive 3D studies of paleontological specimens. Appl Phys Mater Sci Process. 2006;83: 195–202.

[53] Tafforeau P, Smith TM. Nondestructive imaging of hominoid dental microstructure using phase contrast X-ray synchrotron microtomography. J Hum Evol. 2008;54: 272–278. 18045654 10.1016/j.jhevol.2007.09.018

[54] Tafforeau P, Zermeno JP, Smith TM. Tracking cellular-level enamel growth and structure in 4D with synchrotron imaging. J Hum Evol. 2012;62: 424–428. 10.1016/j.jhevol.2012.01.001 22304852

[55] Wilkins SW, Gureyev TE, Gao D, Pogany A, Stevenson AW. Phase-contrast imaging using polychromatic hard X-rays. Nature. 1996;384: 335–338.

[56] Bronnikov AV. Theory of quantitative phase-contrast computed tomography. J Opt Soc Am A. 2002;19: 472–480. 11876309 10.1364/josaa.19.000472

[57] Betz O, Wegst U, Weide D, Heethoff M, Helfen L, Lee W-K, et al Imaging applications of synchrotron X-ray phase-contrast microtomography in biological morphology and biomaterials science. I. General aspects of the technique and its advantages in the analysis of millimetre-sized arthropod structure. J Microsc. 2007;227: 51–71. 17635659 10.1111/j.1365-2818.2007.01785.x

[58] Bronnikov AV. Phase-contrast CT: fundamental theorem and fast image reconstruction algorithms Optics & Photonics. International Society for Optics and Photonics; 2006 pp. 63180Q1–7.

[59] Tafforeau P, Bentaleb I, Jaeger J-J, Martin C. Nature of laminations and mineralization in rhinoceros enamel using histology and X-ray synchrotron microtomography: potential implications for palaeoenvironmental isotopic studies. Palaeogeogr Palaeoclimatol Palaeoecol. 2007;246: 206–227.

[60] Smith TM, Le Cabec A, Bonnin A, Houssaye A, Pouech J, Moggi-Cecchi J, et al. Resolving Pliocene and Pleistocene hominin dental ontogeny with synchrotron virtual histology. American Journal of Physical Anthropology. Calgary, Alberta, Canada; 2014. p. 243.

[61] Smith TM, Tafforeau P, Le Cabec A, Bonnin A, Houssaye A, Pouech J, et al Dental Ontogeny in Pliocene and Early Pleistocene Hominins. PloS One. 2015;10: e0118118 10.1371/journal.pone.0118118 25692765 PMC4334485

[62] Berger LR, De Ruiter DJ, Churchill SE, Schmid P, Carlson KJ, Dirks PH, et al *Australopithecus sediba*: A new species of *Homo*-like australopith from South Africa. Science. 2010;328: 195–204. 10.1126/science.1184944 20378811

[63] Le Cabec A, Tafforeau P, Smith TM, Carlson KJ, Berger LR. Dental development of the *Australopithecus sediba* juvenile MH1 determined from synchrotron virtual paleohistology. American Journal of Physical Anthropology. Calgary, Alberta, Canada; 2014. p. 166.

[64] Le Cabec A, Tafforeau P, Smith TM, Carlson KJ, Berger LR. Dental Development of the *Australopithecus sediba* Juvenile MH1 Determined from Synchrotron Virtual Paleohistology. Proceedings of the European Society for the study of Human Evolution 3. Florence, Italy; 2014. p. 103. Available: http://www.eshe.eu/static/eshe/files/PESHE_3_2014_Florence.pdf

[65] Phong BT. Illumination for computer generated pictures. Commun ACM. 1975;18: 311–317.

[66] Abel RL, Laurini CR, Richter M. A palaeobiologist’s guide to “virtual”micro-CT preparation. Palaeontol Electron. 2012;15: 1–17.

[67] FitzGerald CM. Tooth crown formation and the variation of enamel microstructural growth markers in modern humans. PhD Dissertation, University of Cambridge. 1995.

[68] Schwartz GT, Dean C. Ontogeny of canine dimorphism in extant hominoids. Am J Phys Anthropol. 2001;115: 269–283. 10.1002/ajpa.1081 11424078

[69] Bromage TG, Dean MC. Re-evaluation of the age at death of immature fossil hominids. Nature. 1985;317: 525–527. 10.1038/317525a0 19093314

[70] Schwartz G, Reid D, Dean C. Developmental Aspects of Sexual Dimorphism in Hominoid Canines. Int J Primatol. 2001;22: 837–860. 10.1023/A:1012073601808

[71] Smith TM. Incremental development of primate dental enamel. PhD Dissertation, Stony Brook University. 2004.

[72] Dean MC, Beynon AD, Reid DJ, Whittaker DK. A longitudinal study of tooth growth in a single individual based on long- and short-period incremental markings in dentine and enamel. Int J Osteoarchaeol. 1993;3: 249–264. 10.1002/oa.1390030404

[73] Huda TF, Bowman JE. Variation in cross-striation number between striae in an archaeological population. Int J Osteoarchaeol. 1994;4: 49–52.

[74] Mahoney P. Incremental enamel development in modern human deciduous anterior teeth. Am J Phys Anthropol. 2012;147: 637–651. 10.1002/ajpa.22029 22331636

[75] FitzGerald CM, Rose JC. Reading between the lines: dental development and subadult age assessment using the microstructural growth markers of teeth. Biol Anthropol Hum Skelet Second Ed. 2000; 237–263.

[76] Richards GD, Jabbour RS, Horton CF, Ibarra CL, MacDowell AA. Color changes in modern and fossil teeth induced by synchrotron microtomography. Am J Phys Anthropol. 2012;149: 172–180. 10.1002/ajpa.22103 22729785

[77] Tafforeau P, Le Cabec A, Bonazzi M, Schünemann V, Viola B, Harvati K, et al. Insights about the effect of X-ray imaging on recent fossils: facts, deductions, speculations and phantasms. Proceedings of the European Society for the study of Human Evolution 2. Vienna, Austria; 2013. p. 224. Available: http://www.eshe.eu/static/eshe/files/ESHE_Vienna_2013_Abstracts.pdf

[78] Suwa G, Kono RT, Simpson SW, Asfaw B, Lovejoy CO, White TD. Paleobiological Implications of the Ardipithecus ramidus Dentition. Science. 2009;326: 69–99. 10.1126/science.1175824 19810195

[79] Moggi-Cecchi J, Tobias PV, Beynon AD. The mixed dentition and associated skull fragments of a juvenile fossil hominid from Sterkfontein, South Africa. Am J Phys Anthropol. 1998;106: 425–465. 10.1002/(SICI)1096-8644(199808)106:4<425::AID-AJPA2>3.0.CO;2-I 9712475

[80] Hassett BR. Missing defects? A comparison of microscopic and macroscopic approaches to identifying linear enamel hypoplasia. Am J Phys Anthropol. 2013; n/a–n/a. 10.1002/ajpa.22445 24323494

[81] Reid DJ, Beynon AD, Ramirez Rozzi FV. Histological reconstruction of dental development in four individuals from a medieval site in Picardie, France. J Hum Evol. 1998;35: 463–477. 9774506 10.1006/jhev.1998.0233

[82] Reid DJ, Dean MC. Brief communication: the timing of linear hypoplasias on human anterior teeth. Am J Phys Anthropol. 2000;113: 135–139. 10954627 10.1002/1096-8644(200009)113:1<135::AID-AJPA13>3.0.CO;2-A

[83] Skinner M, Anderson GS. Individualization and enamel histology: a case report in forensic anthropology. J Forensic Sci. 1991;36: 939–948. 1856657

[84] Yamashita Y, Ichijo T. Comparative studies on the structure of the ameloblasts Mechanisms of tooth enamel formation Part I—Cytology and Cytochemistry of Forming Cells. Tokyo: Quintessence; 1983 pp. 91–107.

