# Supplementary figures and images for: Accessing Developmental Information of Fossil Hominin Teeth Using New Synchrotron Microtomography-Based Visualization Techniques of Dental Surfaces and Interfaces
Source: PLoS One. 2015 Apr 22;10(4):e0123019. doi: 10.1371/journal.pone.0123019 (PMC4406681; doi:10.1371/journal.pone.0123019)

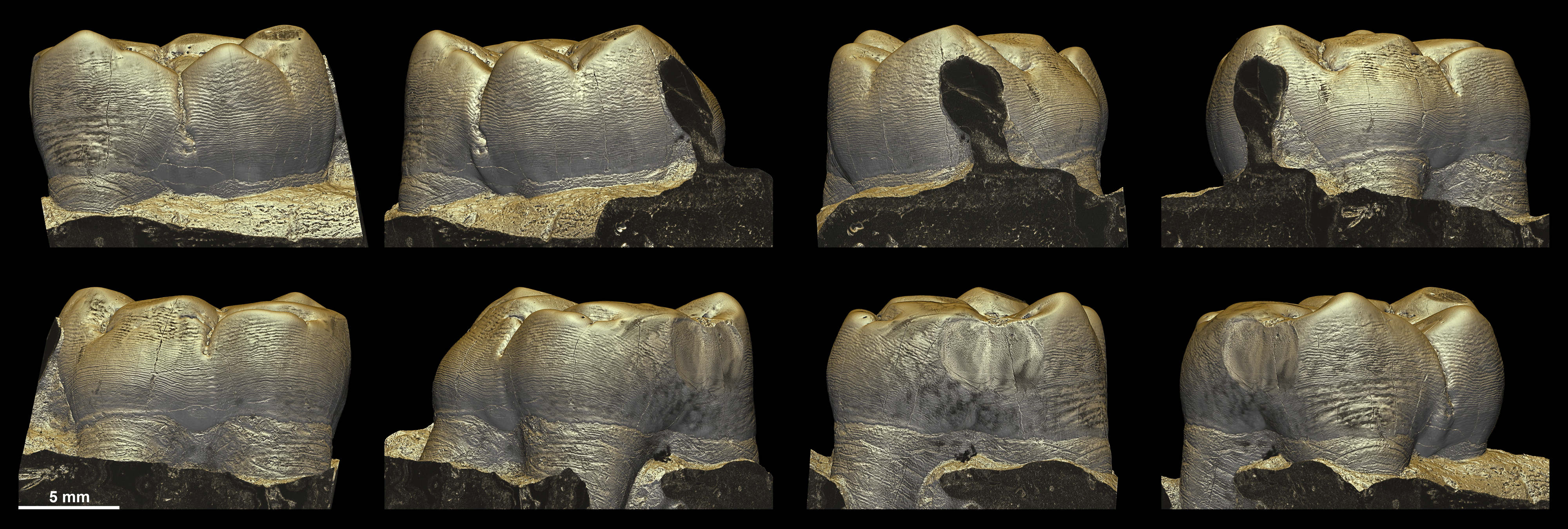

Supplement: S1 Fig — The LLM1 of MLD2 imaged every 45° along the transverse axis following rendering by the protocol detailed here. This allows visualization of developmental defects or long-period features around the tooth to ascertain their authenticity (and rule out imaging or reconstruction artifacts). File name: S1_Fig.tif. (TIF) [file pone.0123019.s001.tif]

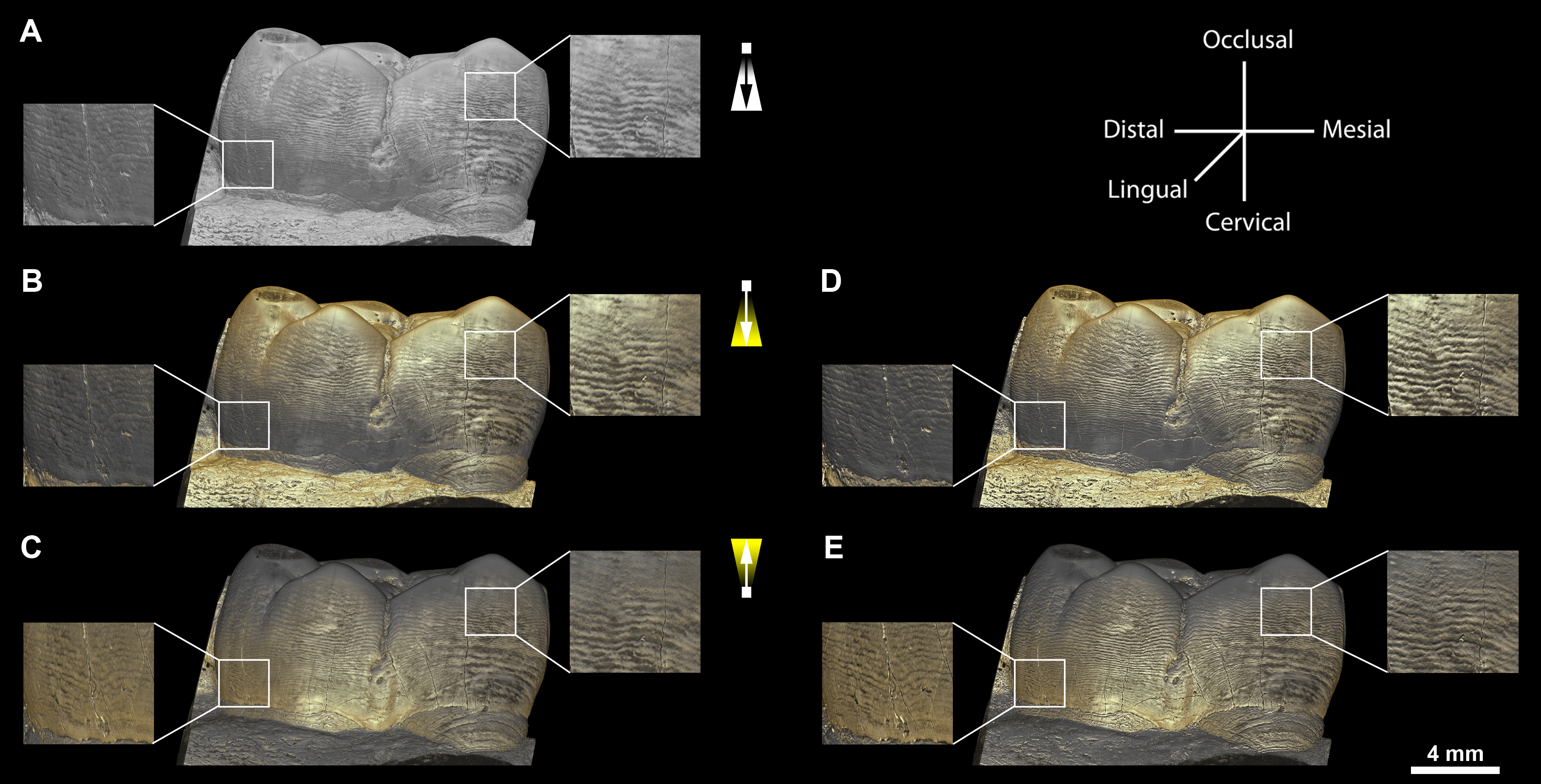

Supplement: S2 Fig — Segmented enamel surface of the MLD2 LLM1 rendered in 3D using Phong’s algorithm. First, the model is lit with the two default light sources (the first is oriented perpendicular to the screen and coming towards the user, while the second is coming from the top) with all components having a white hue (A). Then, the second light source is successively oriented from the top (B) and bottom (C) with a low white ambient component, a moderate orange diffuse component, and a pale blue specular light. Two masks of reinforcement are then successively computed in Photoshop from the high frequencies of the top view (B) and the bottom view (C). These masks enhance the high frequencies of the views lit from the opposite direction: i.e., the mask computed from (B) enhances the high frequencies of (C) resulting in (E). The same is true for the mask computed from (C) reinforcing (B) and resulting in (D). Microscopic increments on the outer enamel surface (perikymata) are shown in corresponding insets of each lighting condition. File name: S2_Fig.tif. (TIF) [file pone.0123019.s002.tif]

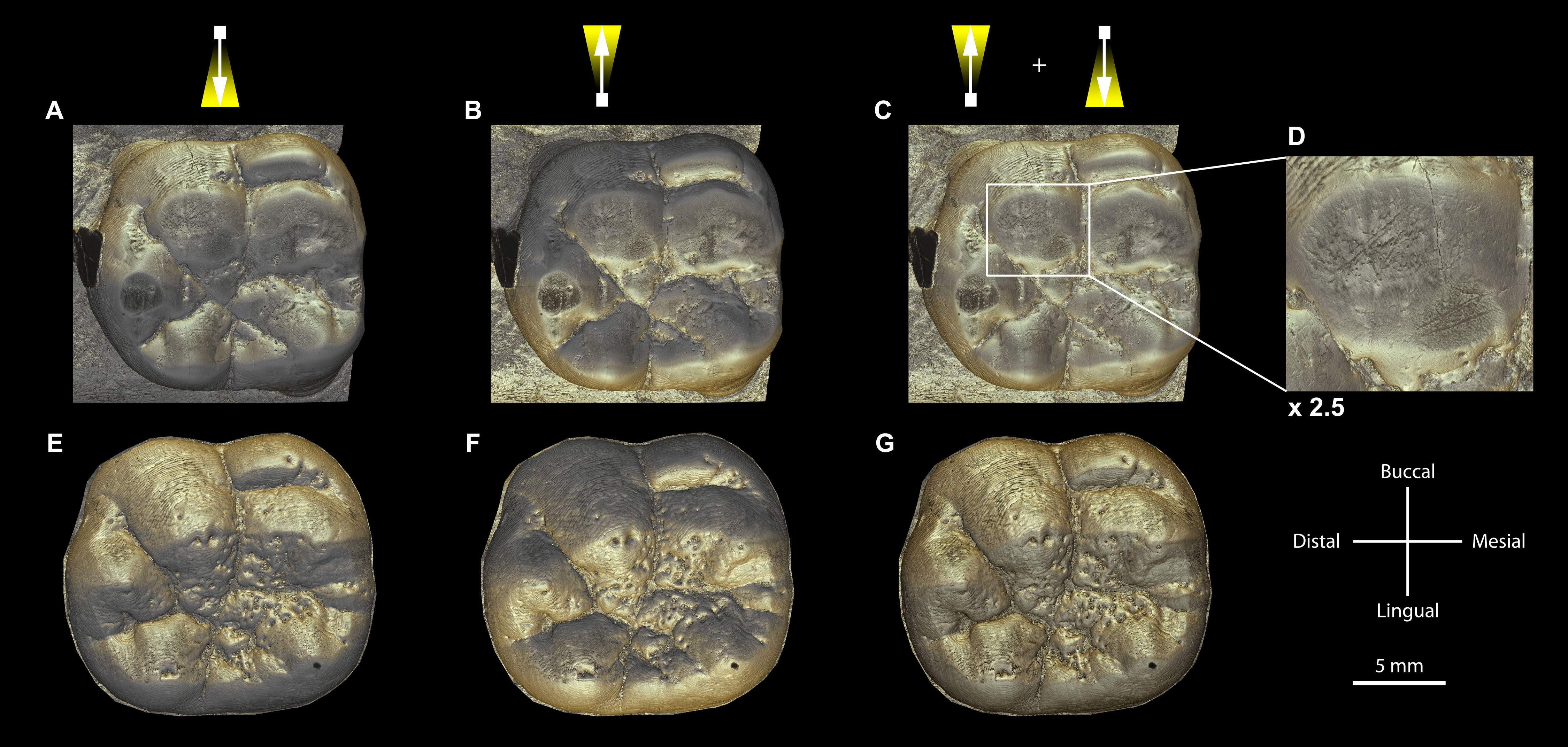

Supplement: S3 Fig — The LLM1 shows occlusal wear facets, macro- (A-C) and microwear (D) features. The unworn occlusal morphology of the LLM2 displays perikymata from the cusp tip downwards along the lateral enamel (E-G). File name: S3_Fig.tif. (TIF) [file pone.0123019.s003.tif]

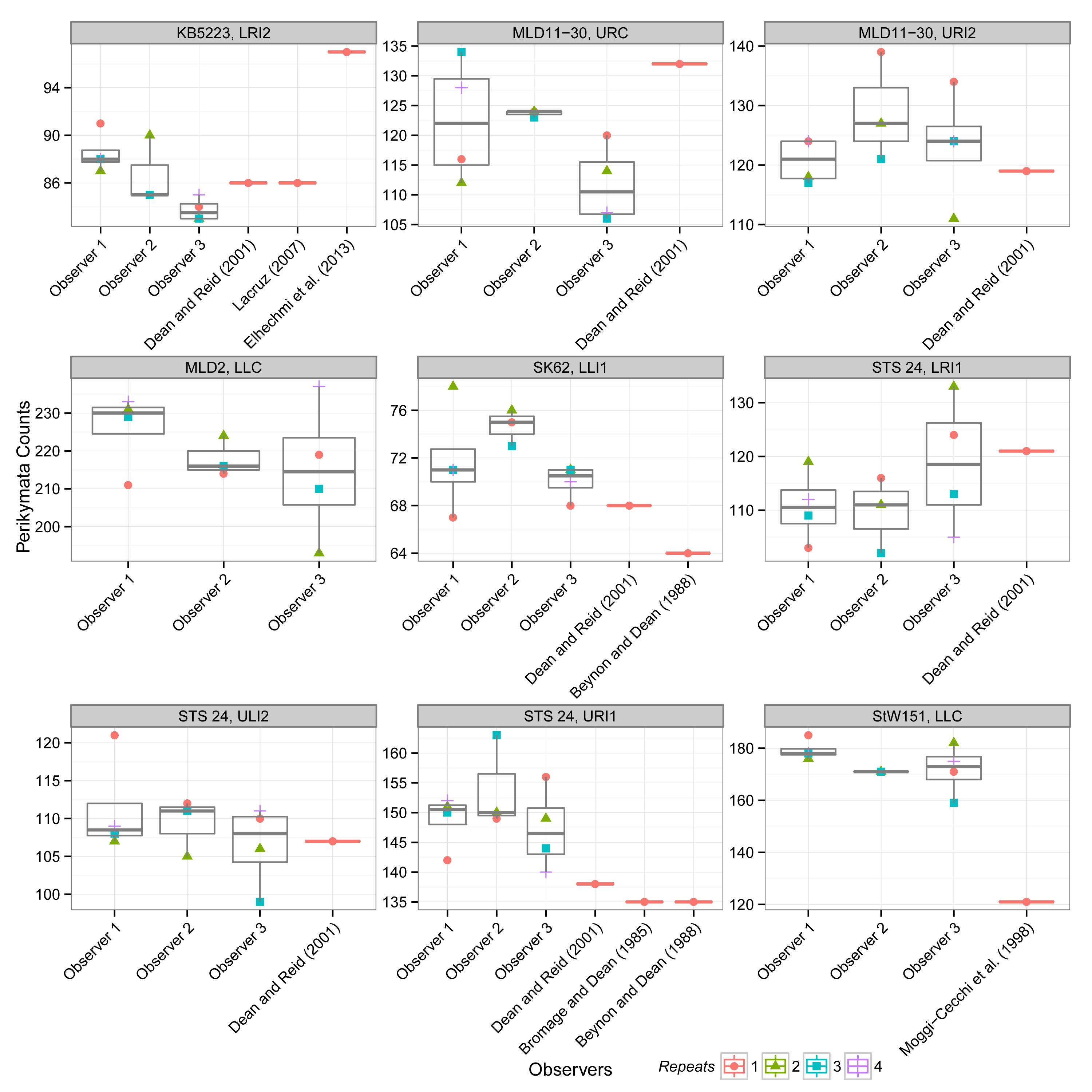

Supplement: S4 Fig — Whisker plot showing the variability in the perikymata counts for each observer, and the overall variability among observers, compared to perikymata counts published in the literature. File name: S4_Fig.tif. (TIF) [file pone.0123019.s004.tif]

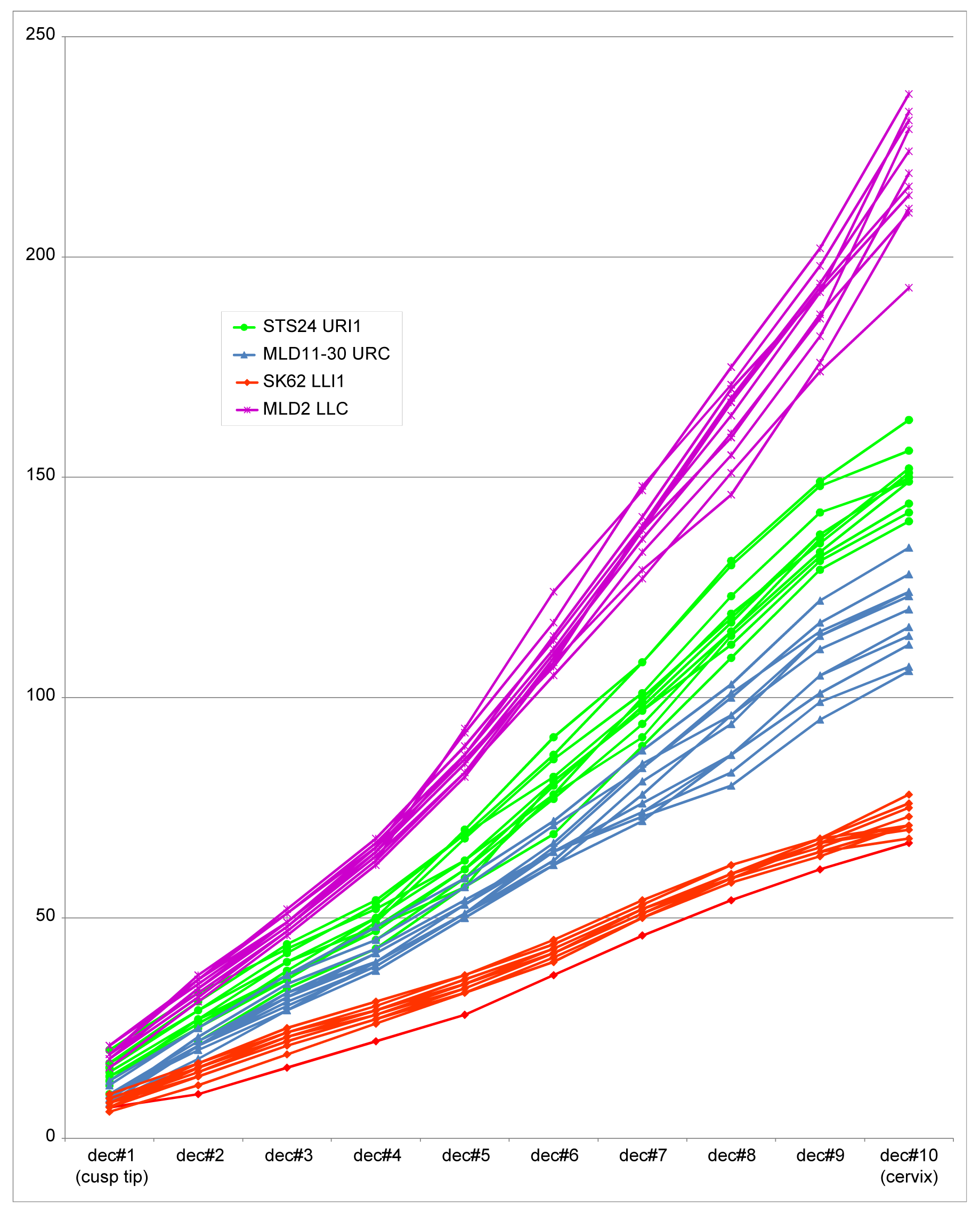

Supplement: S5 Fig — Cumulative plot of perikymata counts of all observers, per decile of crown height, for four teeth (STS24 URI1, MLD11-30 URC, SK62 LLI1 and the unerupted MLD2 LLC). ‘dec#1’ designates the first decile at the cusp tip, down to ‘dec#10’ at the cervix. File name: S5_Fig.tif. (TIF) [file pone.0123019.s005.tif]

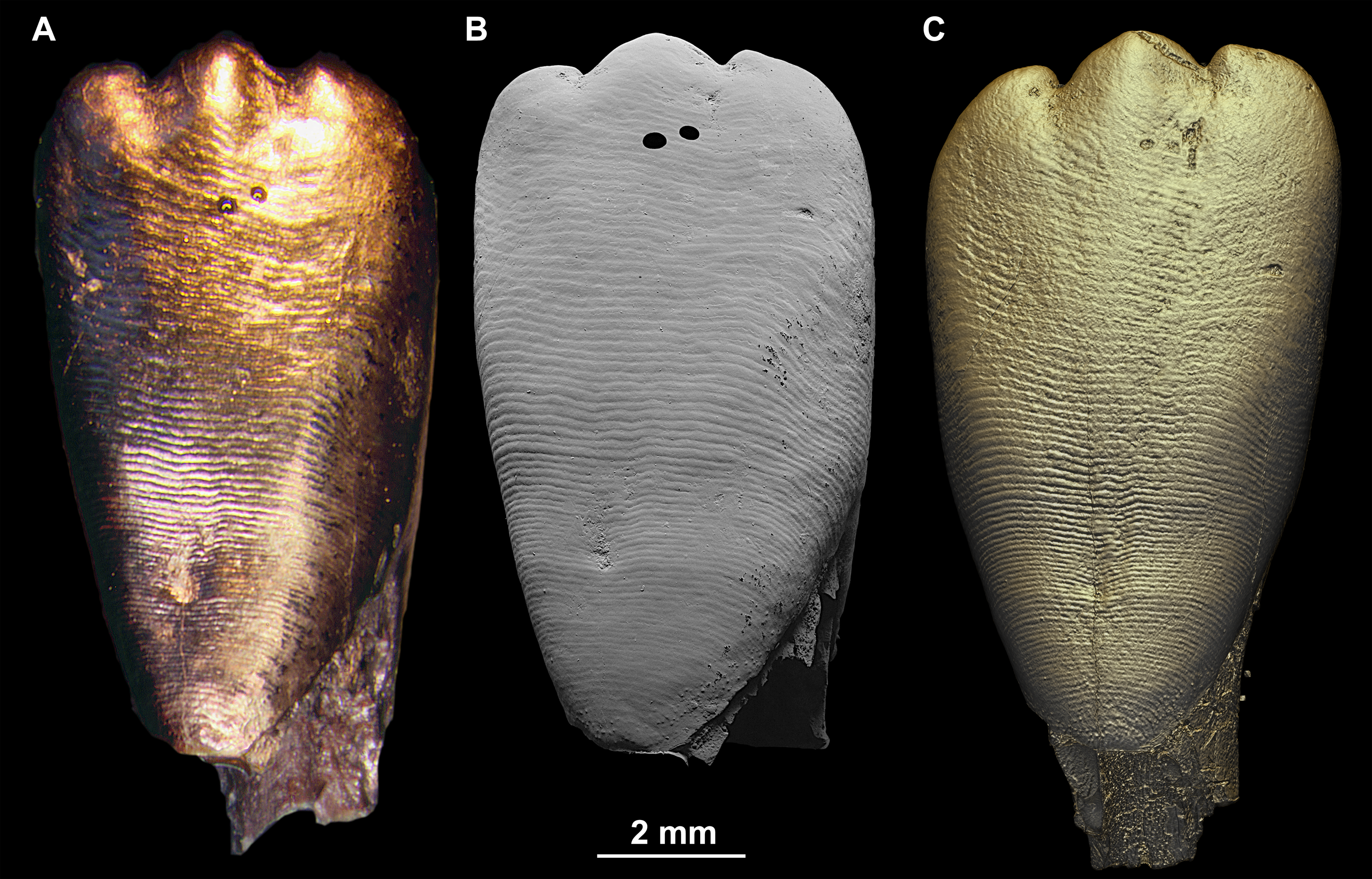

Supplement: S6 Fig — Comparison of imaging techniques for perikymata visualization on the labial surface of the LRI2 of KB5223, with (A) stereomicroscope, (B) SEM, and (C) 3D rendering using PPC-SRμCT data (Phong, colored lights and normalize gradient in VGStudio MAX 2.2). File name: S6_Fig.tif. (TIF) [file pone.0123019.s006.tif]

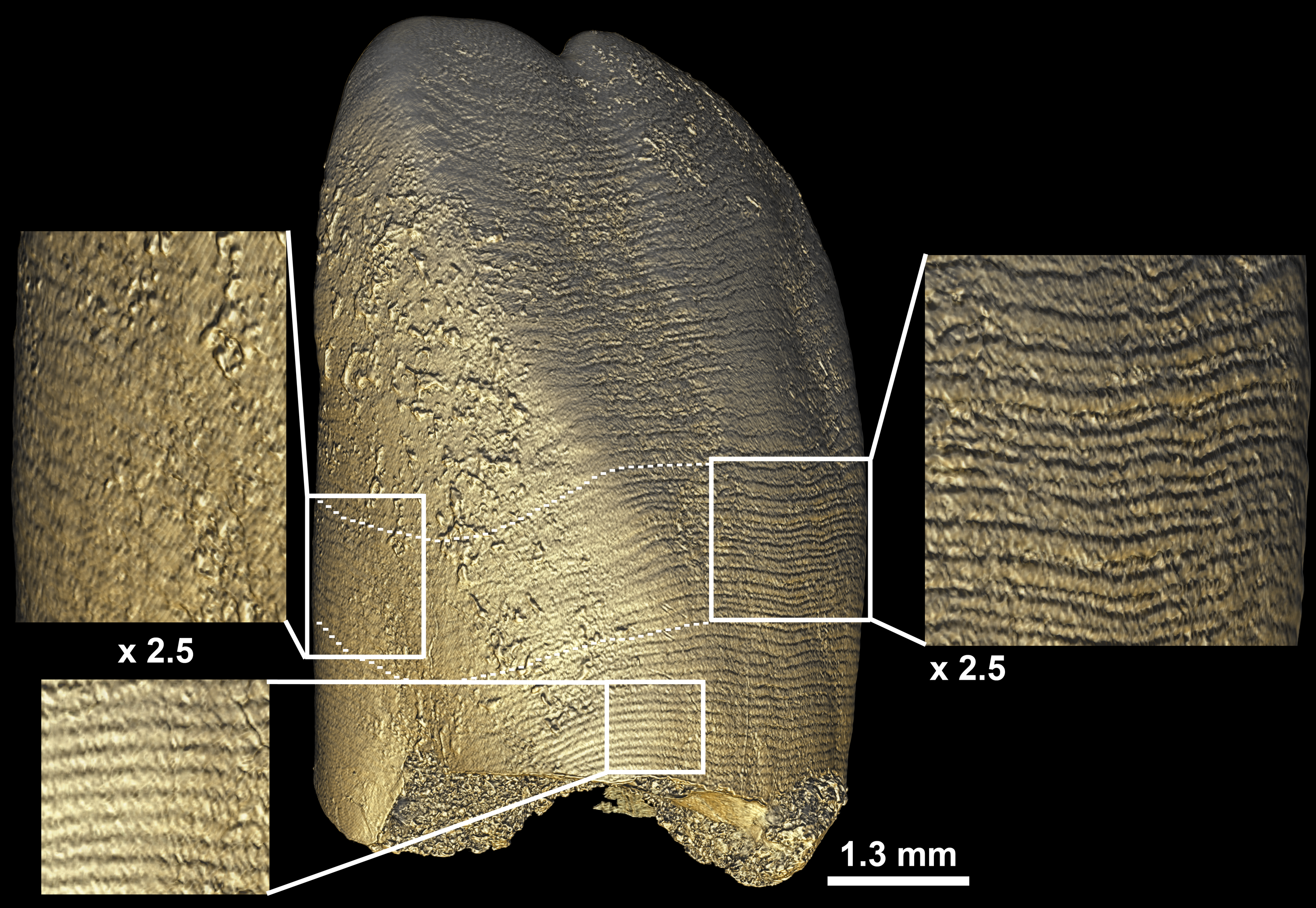

Supplement: S7 Fig — Three-dimensional model of the LLI2 of KB5223 (light source 2 is oriented from the top) showing subdivisions of perikymata (right inset). This phenomenon is only local, since there are no subdivisions for a contemporaneous area of the crown surface (top left inset). It is also not consistent through time, because there are no subdivisions in the cervical part of the crown (bottom left inset). S8 and S9 Figs reveal that this phenomenon can be induced by demineralization of the enamel sub-surface. See S1 Supporting Information (section II) for a discussion. File name: S7_Fig.tif. (TIF) [file pone.0123019.s007.tif]

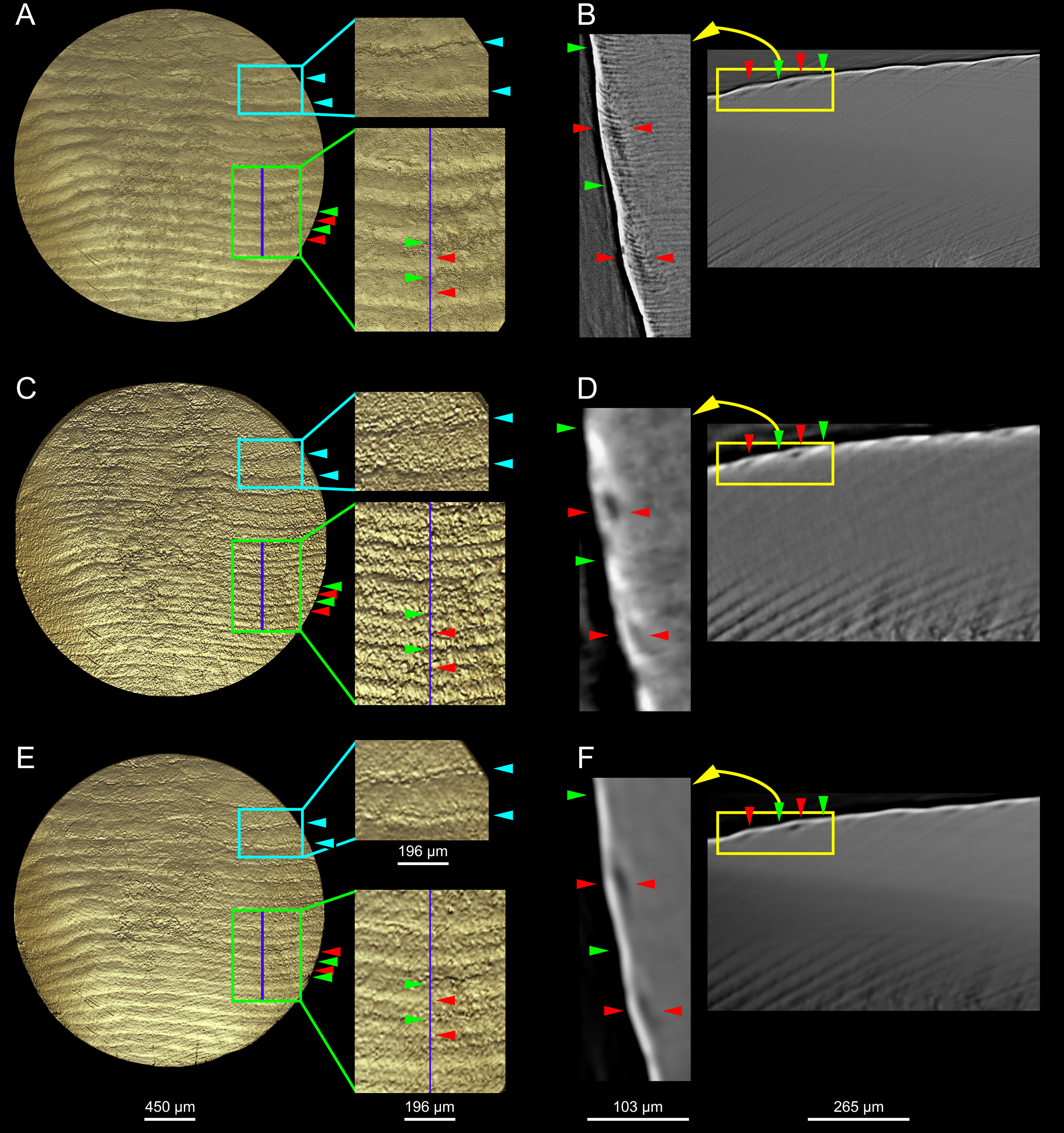

Supplement: S8 Fig — Impact of tomographic partial volume effect on demineralized enamel sub-surface in the labial aspect of the KB5223 LLI2. The same region of interest is imaged at high resolution (0.7 μm voxel size, top row, A and B), 5 μm (middle row, C and D) and degraded 0.7 μm (bottom row, E and F) in an area showing perikymata/Retzius line subdivisions of equal size (green frame) on the 3D models (C and E) and the corresponding 2D virtual slices (location shown as a blue line on the 3D): 100 μm-thick (right) and inset showing the 5 μm-thick slice (left from the yellow frame). The turquoise inset illustrates the thin sheet of enamel overlaying the perikymata ridges, and which are not an artifact as it is also visible using other imaging techniques (see S6 Fig). Green arrows materialize the location the “true” long-period lines, while red arrows show the location of the subdivisions (not existing on A). File name: S8_Fig.tif. (TIF) [file pone.0123019.s008.tif]

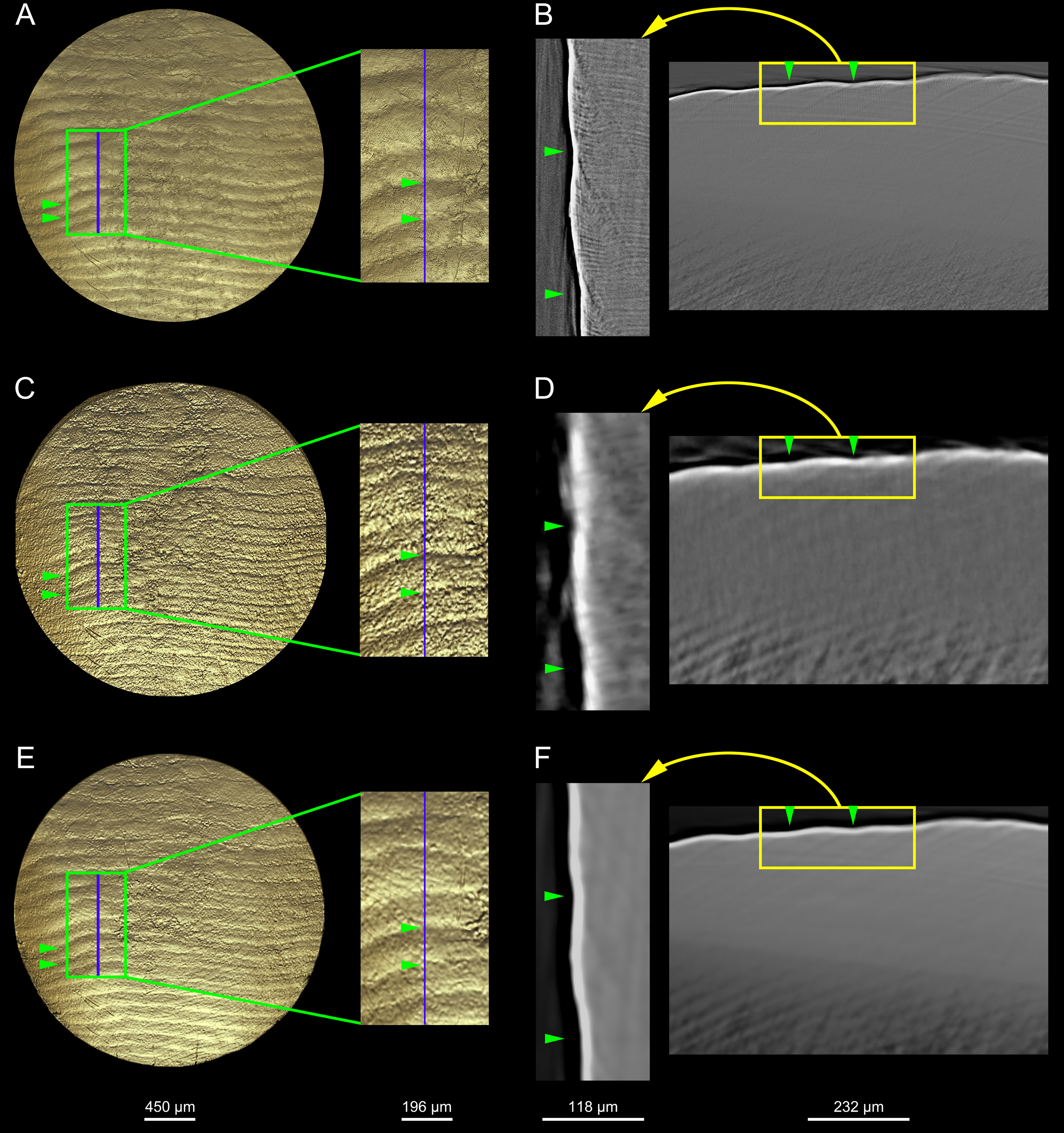

Supplement: S9 Fig — Imaging of a portion of the labial aspect of the KB5223 LLI2 crown not showing demineralized long-period line subdivisions, corresponding to same time period than the area presented in S8 Fig. Same configuration and color-coding as for S8 Fig (save for the turquoise inset that is not shown). It shows that the perikymata subdivisions presented in S8 Fig are induced by the partial volume effect when the resolution is not high enough to fully discriminate the external surface and the sub-surface demineralization. File name: S9_Fig.tif. (TIF) [file pone.0123019.s009.tif]

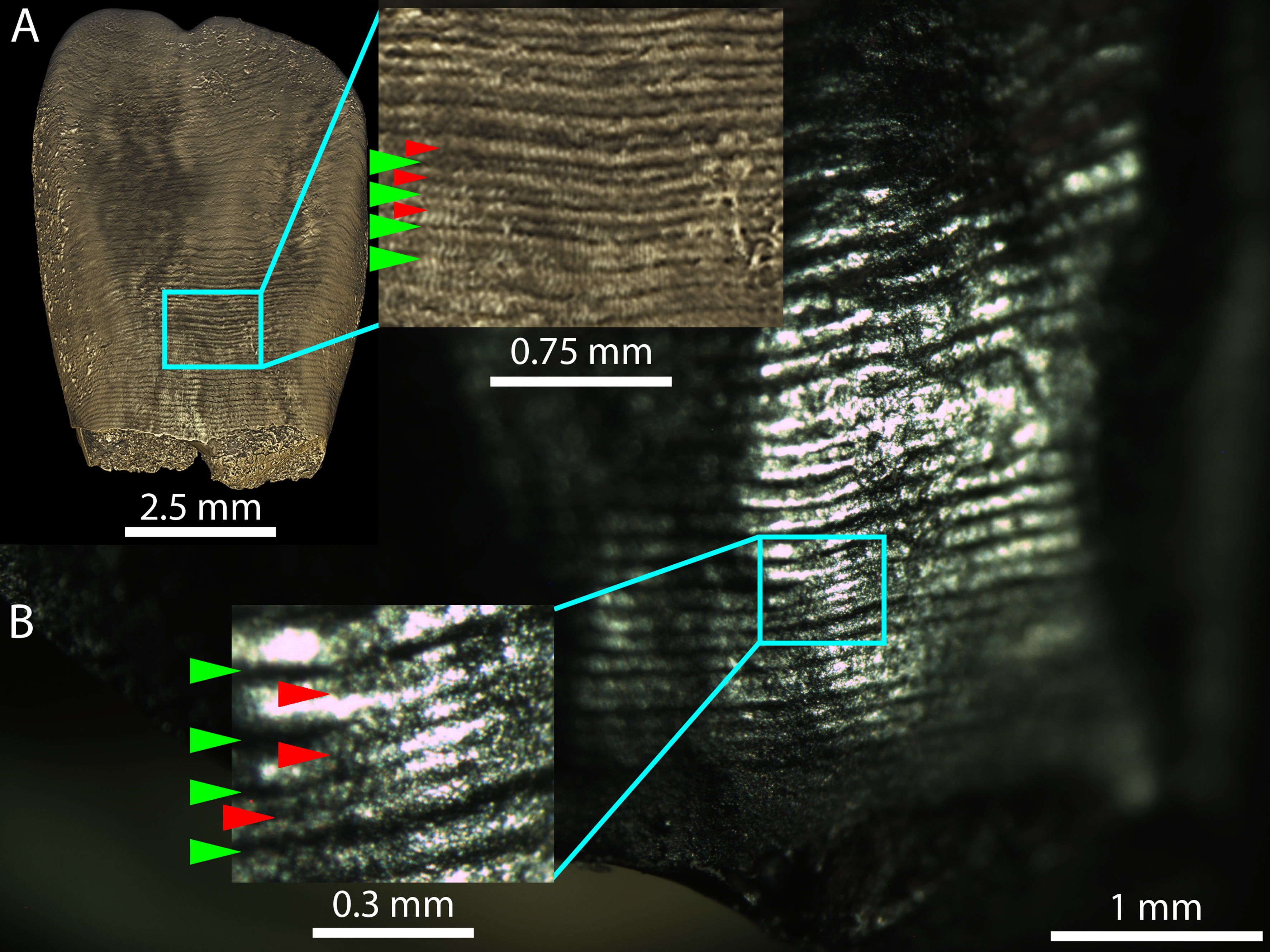

Supplement: S10 Fig — Perikymata subdivisions non-related to imaging artifacts are visible on the PPC-SRμCT-based 3D model of the KB5223 LLI2 (A, LS2 oriented from the bottom) and on the area framed in turquoise (in [A]) on the labial aspect of a silicone cast of the same tooth, observed under a binocular microscope (B). This illustrates the case where the demineralization of the enamel subsurface reaches the surface and creates a visible structure on the outer enamel surface. Green arrows point to true anatomical perikymata and red arrowheads show the subdivisions. The binocular picture is a courtesy of Dr. Tanya M. Smith. File name: S10_Fig.tif. (TIF) [file pone.0123019.s010.tif]

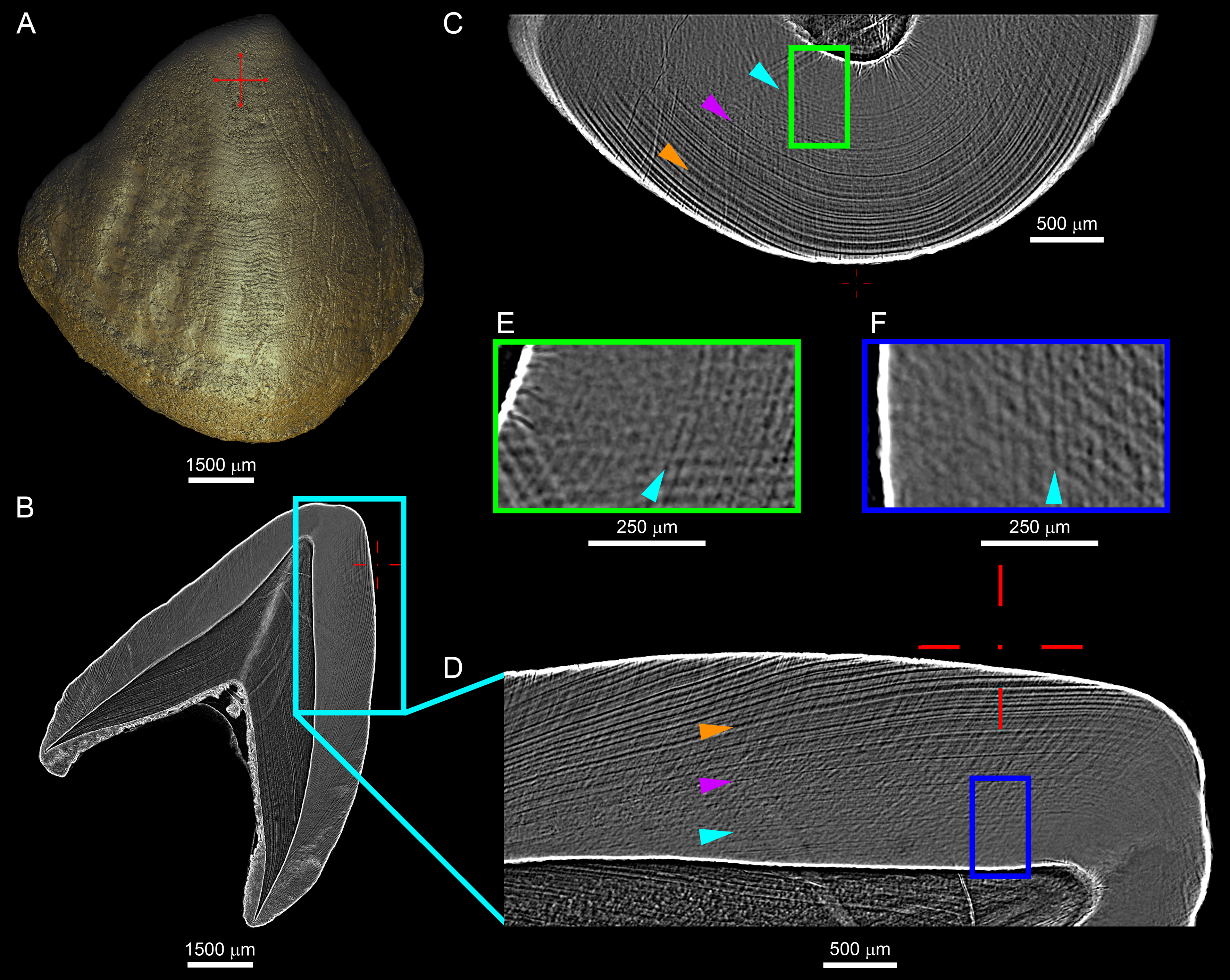

Supplement: S11 Fig — The red cross illustrates where the measurement has been taken on the 3D model of the STS2 ULC (A), on 200 μm-thick sagittal (B) and transversal (C) slices in the cuspal enamel. Colored arrows show corresponding accentuated lines on both the transversal slice (C and E) and on magnified zone (D and F) from of the sagittal slice. Retzius lines are easier to follow as concentric lines on the transversal slices (C and E), and their tracing can be matched back onto the sagittal slice (B) for measuring cuspal distances to calculate the CuDSR. It has to be noted that C, D, E and F were enhanced with an unsharp filter in Adobe Photoshop CS4. File name: S11_Fig.tif. (TIF) [file pone.0123019.s011.tif]

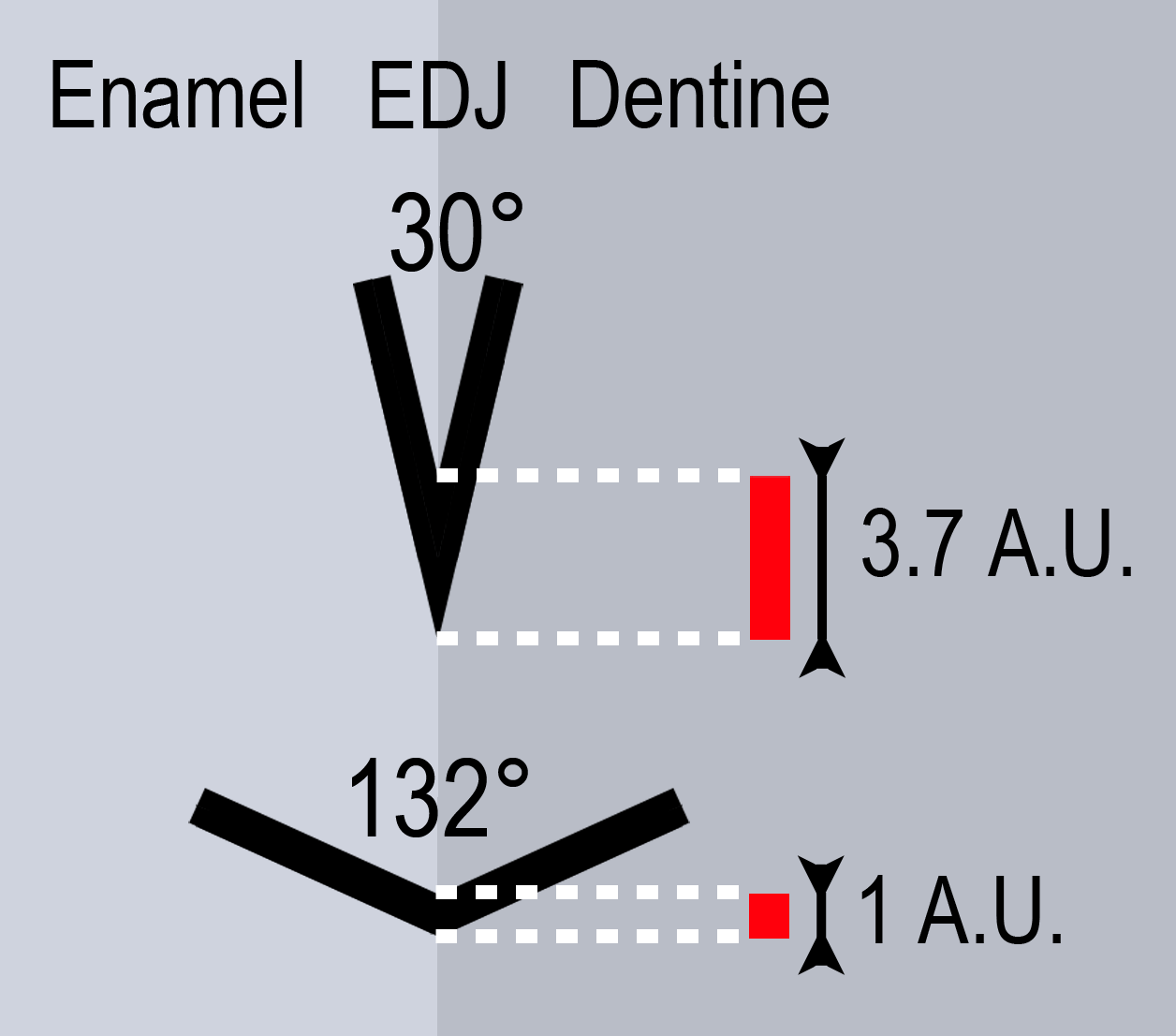

Supplement: S12 Fig — Illustration showing the importance of the angle of intercept of the long-period growth lines and developmental defects at the EDJ. A stress reaching the EDJ with an acute angle (here 30°/2) will manifest as a thicker band (3.7 A.U.; ‘A.U’ stands for ‘arbitrary unit’) than in the case of a larger angle (here 132°/2 corresponding to a thickness of 1 A.U. on the EDJ). A stress occurring early in dental development, such as the neonatal line in the permanent first molar, will be very strongly tangent to the EDJ because of the high extension rate during early cuspal enamel and dentine formation. This will result in a large band on the dentine horn tip. File name: S12_Fig.tif. (TIF) [file pone.0123019.s012.tif]

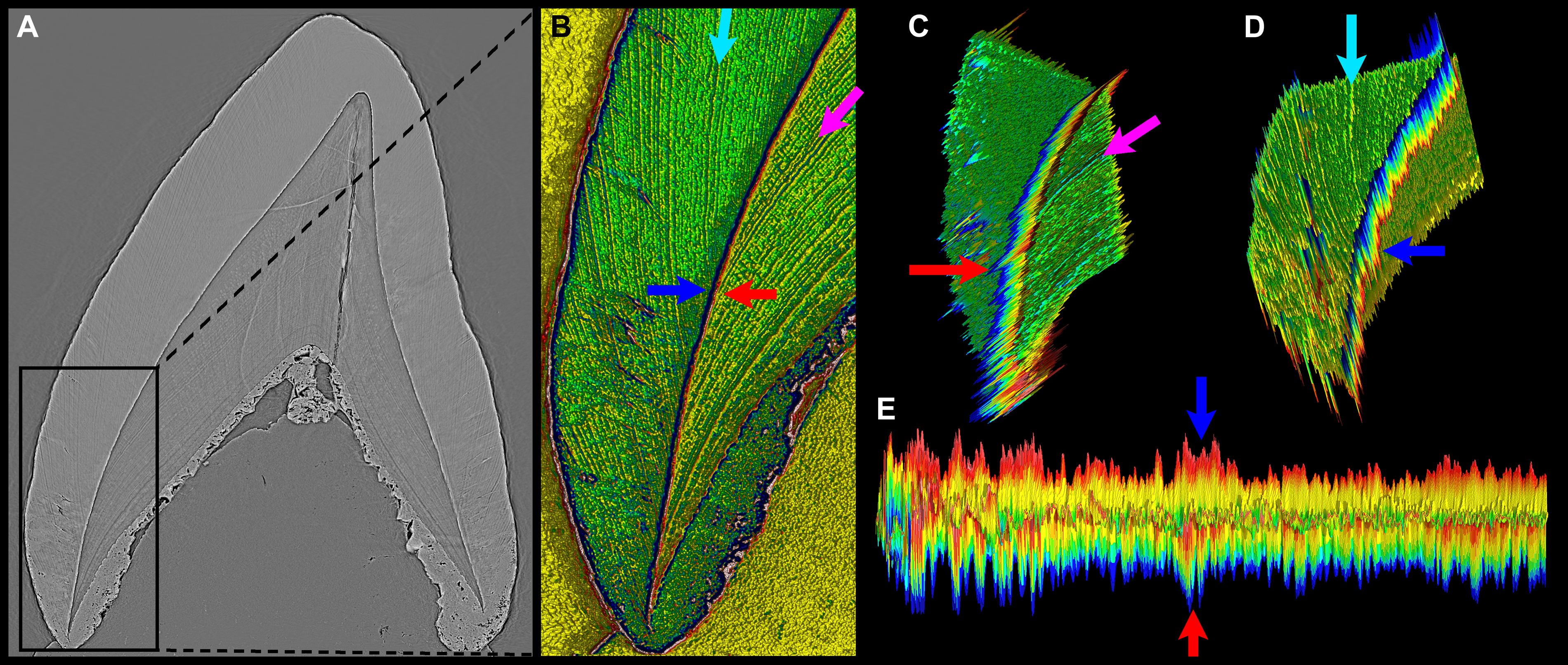

Supplement: S13 Fig — Virtual 5 μm slice of the STS2 ULC through the PPC-SR-μCT dataset reconstructed in edge detection, where phase contrast reveals incremental long-period growth lines and a stress pattern (A). An inset shows the incremental growth lines in enamel and dentine, and developmental defects meeting at the EDJ (B). In the 3D plots shown in (B-E), both the surface relief (peaks) and coloring (“Spectrum” Look Up Table often used for topographical coding in Geographical Information System) represent the intensity of the gray values in both the black and white fringes, similar to topographic elevation. The higher the peak, the brighter the gray value will be in the white fringe (up to dark blue) or the darker the gray value in the black fringe (up to red). For the same portion of the tooth, for the white (C) and the black (D) fringes are shown as isolated. A detail of the white (above) and black (below) fringes at the EDJ (E) showing the peaks for the gray values levels where stress lines, generally of lower densities, reach the EDJ (color-coded arrows also shown in B-D; red for the black fringe and blue for the white fringe). These peaks in gray levels yield stress patterns that may be read as a “barcode” on the segmented 3D dentine models, and are visualized as bright or dark stripes of various width using Phong 3D rendering and colored light sources. File name: S13_Fig.tif. (TIF) [file pone.0123019.s013.tif]
