# Supplementary material for: Accessing Developmental Information of Fossil Hominin Teeth Using New Synchrotron Microtomography-Based Visualization Techniques of Dental Surfaces and Interfaces
Source: PLoS One. 2015 Apr 22;10(4):e0123019. doi: 10.1371/journal.pone.0123019 (PMC4406681; doi:10.1371/journal.pone.0123019)
Supplement: S1 Supporting Information — File name: S1_Supporting_Information.docx. (DOCX) [file pone.0123019.s016.docx]

**S1 Supporting Information**

1. ***Perikymata Counting: Intra- / Inter-Observer Agreement Study and Comparison between Imaging Techniques***
   1. ***Perikymata counting: inter- / intra-observer agreement study***
      1. ***Protocol***

We assessed the reliability of perikymata counting by undertaking an inter-/intra-observer agreement study to test whether the 3D renderings (Phong and combination of colored lights) allowed observers with different levels of experience to consistently visualize and identify perikymata. We selected teeth for which previously published values were available (i.e., all teeth mentioned in S1 Table belonging to SK62, STS24, MLD11-30 and the LRI2 of KB5223). We also included the permanent mandibular left canine from MLD2 and StW151, which have never been counted before. These latter two teeth will serve as case-studies since we will calculate their crown formation time from the data obtained with the techniques presently shown. Counting and tracing the enamel long-period lines were performed in Photoshop. Observations were made several times, on high quality plates comprising eight views (rotation every 45°) of the selected teeth (e.g., S1 Figure for the LLM1 of MLD2, which is not included in this part of the study), and with a minimal interval of one day between counting sessions of the same tooth by the same observer, to avoid observation inter-dependency. To count perikymata on the labial side of the tooth of interest, the three observers used the other views of the tooth to check structural details in locations where the perikymata are faint or difficult to identify. Looking at both the top and bottom orientations of LS2 helped identify the presence and location of perikymata. Following the definition of perikymata [1,2] as waves, we counted the ridges and drew their limits in the grooves. For each tooth, we first drew lines where we identified inter-perikymata grooves directly on the plate with the eight views (via layers in Photoshop), and then we counted the number of perikymata on the whole height of the crown.

In addition, we further divided this count into deciles of the crown height (decile #1 is at the cusp tip and decile #10 is at the cemento-enamel junction) for comparisons with published data, and also to identify locations where inter- and intra-observer variability is maximal. To do so, in the file of the plate containing the eight views of the 3D models, we drew in a separate layer a ladder sub-divided into ten equidistant intervals onto the labial view of the tooth, spreading from the cusp tip to the cervical most point of the labial side of the tooth in the mid-sagittal plane. We are aware that this technique does not follow the curved surface of the crown, which would require intervening directly on the 3D model in VGStudio MAX 2.2. However, we think that the way we designed our deciles is fairly comparable to the technique used by Dean and Reid [3], involving measuring the buccal height of the crown at right angles to the optical view of the microscope, which is then divided into ten equal parts. In addition, we do not think it is possible to draw a curved line divided into ten equal segments, and we could not have used the drawing and counting tools that are available in Photoshop. To avoid inter-observer error, the ladder was positioned and scaled following the crown size of each tooth (using isometric scaling) by one of us (ALC), and this template file was duplicated for each observer, and each observation. This rules out any possible source of error related to the definition of the deciles. We hypothesized that the highest variability would be at the cusp tip, because it is sometimes polished and has perikymata that are fainter and more spaced, and at the cervix, where perikymata are more packed and enamel is often less well preserved. We reported decile counts primarily to see where would the major variation be among observers counting sessions, and then for a qualitative comparison with (scarce) published data on these specimens.

- - 1. ***Statistics***

Because of our small sample size and the limited number of counting sessions, we only report descriptive statistics computed in Microsoft® Office Excel® 2007 and R 2.15.0 [4,5] for the full counts, to illustrate the consistency for each observer and among all observers. To test whether the variability between observers could affect our observations, we performed a single factor ANOVA test (in Excel) for which we pooled together all the deciles of all the teeth for all observers’ counting sessions. In addition, we plotted the cumulative values of counts across deciles for a few teeth to illustrate the degree of consistency of the counts and to highlight the regions of the crown where counts showed the largest variability.

- - 1. ***Results***

Results (average counts per observer) and comparisons to the literature are provided in S3 Table. The agreement among the three observers was reasonably good (S4 Figure). Indeed, the results of the single-factor ANOVA analysis yield no significant results (for each observer, all deciles for all teeth were pooled together, *p*>>0.01, tab “ANOVA-single-factor” of S2 Supporting Information), showing that the difference between the variances of the three observers has no significant impact.

The SK62 and KB5223 incisors were the clearest teeth to count, yielding maximal agreement within and between observers, with a maximum difference of five perikymata among mean counts (S2 Supporting Information). Perikymata on these teeth were clearly visible and easily identified, although they were fainter in the cervical-most area for SK62. S2 Supporting Information (Tab “Average deciles”) shows the differences between the published values by Dean and Reid [3] and the mean of all of our observations for all observers. The SK62 and KB5223 incisors show little deviation from the published values (±1-2 perikymata).

Despite strongly marked perikymata (Fig. 1) the MLD11-30 canine has a high level of perikymata subdivisions (see below S1 Supporting Information, section II) on the cervical half of the crown (Fig. 4), and this made the identification of perikymata more ambiguous (a maximum of 12 perikymata difference between the observers’ mean counts), despite the fact that the three observers used the other views to assess whether a groove was encircling the tooth or not. This resulted in a rather large difference with published values, which exceed ours by six perikymata (Tab “Average deciles” of S2 Supporting Information). The lateral incisor of MLD11-30 showed fewer cases of perikymata subdivisions (maximum difference of eight perikymata between the observers’ mean counts).

The STS24 teeth had an intermediate level of difficulty (discrepancies from five to nine perikymata), with polished cusp tips and disturbed cervical areas. In general, the agreement between observers with different levels of experience is reasonably good.

Overall, when compared with published values, our perikymata counts are roughly similar for STS24 ULI2 and KB5223 LRI2 [3,6], higher for MLD11-30 URI2, SK62 LLI1 and STS24 URI1 [3,7,8] and lower for MLD11-30 URC and STS24 LRI1 [3]. We counted 86 perikymata on average for all observers for the KB5223 LRI2, as did Dean, Reid, and Lacruz [3,6], this is expected for teeth where structures are “easily” identifiable. Elhechmi *et al.* [9,10] developed an automated protocol for perikymata counting based on an optical device. They reported 97 perikymata for the KB5223 LRI2, which is 11 perikymata more than both an average of our repeated counts and previously published values. Since their quantification was done along a profile on the whole height of the crown, we interpret this difference as stemming from the detection by the optical device of some of the split perikymata that are particularly marked along the vertical mid-line (cusp tip to middle of cervical base) on the labial aspect of the crown.

Details of the decile counts are presented and analyzed for each tooth in the same named tabs of S2 Supporting Information. We found a reasonable agreement within and between observers, although the cervical area shows a maximum of variability (S5 Figure, and tabs in S2 Supporting Information for each tooth investigated). Although this is not so clear-cut for all the teeth, there seems to be a trend towards higher counts towards the cusp tip for our three sets of observations versus the published values (see multiple tabs in S2 Supporting Information). The situation is less clear for the cervix. In some teeth, our counts exceed those of the literature (e.g., STS24 LRI1, MLD11-30 URC and SK62 LLI1), while for other teeth we reach a lower number (e.g., STS24 ULI2 and MLD11-30 URI2). However, the teeth with subdivisions of perikymata seem to generate more uncertainty and more variation in the number of perikymata identified.

The fact that we did not use a curved line to subdivide the crown height into deciles is a common drawback with classical methods, although our technique has the advantage over SEM or binocular microscopy that there is no distortion of the image of the crown (due to the principle of tomographic reconstruction itself). Indeed, Dean and Reid, [3] acknowledged that counting perikymata and defining deciles on tooth casts under a binocular microscope can yield distortion especially for the “bulbous cervix” of early hominin canines. In future research, an approach based on semi-landmarks could be used to define the mid-sagittal line of the buccal surface of anterior teeth, directly on the 3D model of the tooth, this crown height could be subdivided into 10 equal segments, and perikymata could be after counted on a high resolution 3D pdf of this surface including the tooth and the deciles. This would yield an accurate quantification of perikymata count per decile.

- 1. ***Comparison between stereomicroscope, SEM and 3D renderings***
     1. ***Limitations of each technique***

Despite easy access and quick use, stereomicroscopes do not provide a focused image of the whole surface of the tooth crown (except with a Z-series of focal planes combined with an algorithm to integrate information in focus). High quality imaging of actual teeth is very difficult due to the translucency of enamel, and observations are performed on high resolution casts of fossil specimens derived from silicone molds. Repetitive molding (for various research purposes) can be detrimental to brittle specimens, and traces of the molding material often remain entrapped in the small cavities and fissures.

Dean imaged the labial surface of the MLD11-30 incisor [11: p. 3398, Fig. 1a] using a SEM, revealing perikymata and hypoplasias. In most cases, SEM also requires use of casts, and the samples have to be further coated with metal for electron evacuation during imaging. Scanning electron microscopy is an efficient way to produce high resolution [down to the nm scale: 12] and high quality images for perikymata counting, but it induces a geometrical distortion [e.g., 13,14] when imaging relatively large objects such as teeth, prohibiting reliable quantification of perikymata distribution. This can however be minimized by acquiring several small regions of interest that can be later combined into a mosaic image of the whole tooth surface. The tooth cannot however be visualized at 360° using the same tooth position and lighting system, as it is with synchrotron data.

PPC-SR-µCT yields external and internal sources of information from dental tissues (e.g., cuspal enamel thickness, internal stress patterns, long-period line periodicity). For a discussion about some limitations of this technique, see here below S1 Supporting Information - section II (possibility of artifactual perikymata subdivisions) and the last paragraph of the part called “New techniques for long-period growth line visualization” in the Discussion of the main manuscript.

- - 1. ***Protocol***

For an exploratory comparison between imaging techniques, a high resolution mold and cast of the KB5223 LRI2 was prepared with Struers Repliset and epoxy resin (Epo-Tek 301, Epoxy Technology, [www.epotek.com](http://www.epotek.com)). After curing, the labial side of the cast was sputter-coated with gold and examined with a Tescan Vega3 SEM using a topographical backscattered detector, at the Wyss Institute for Biologically Inspired Engineering at Harvard University. The SEM image was obtained from a single shot. In addition, the cast was photographed under a stereomicroscope (Leica MZ12.5), coupled to a Canon EOS550D reflex numerical camera, under different light intensities and orientations for optimizing the visibility of the perikymata on the whole crown surface. Six different lightings were combined in Photoshop to generate the final picture showing perikymata on the whole surface of the tooth.

Perikymata in these additional images were counted only once as the aim of this imaging comparison was to demonstrate that the synchrotron results correspond to real dental structures that are not obscured by artifacts from strong propagation phase contrast. We did not make the decile counts. Indeed, due to the geometry of the SEM system (and possibly of the cast shape after many handlings), there is a strong distortion of the picture. This distortion exists only to a limited extent in stereomicroscopy and does not exist in 3D models generated from synchrotron scans (reconstruction from parallel projections). A correction of this distortion produced by the SEM geometry would require using an external calibration system, which is difficult to apply for an object with a topology as complex as that of a tooth. An alternative would be to image small portions of the crown following a grid with displacement of the sample below the vertical axis of the electron beam and then concatenate them into an image free of distortion. Although having no effect on total perikymata counts, this distortion prevents any reliable count per deciles.

- - 1. ***Results***

S6 Figure shows a comparison between an SEM image (obtained from a single imaging angle), an image produced with a stereomicroscope using multiple light combinations and one of our 3D model (Phong and colored lights) for the LRI2 of the KB5223. Two observers counted 83 and 97 perikymata on the SEM image, and 86 and 87 on the stereomicroscopic image.

- 1. ***Literature cited***

1. Risnes S. Rationale for consistency in the use of enamel surface terms: perikymata and imbrications. Eur J Oral Sci. 1984;92: 1–5.

2. Preiswerk G. Beitrage zur Kenntnis der Schmelzstruktur bei Saugetieren mit besonderer Beriicksichtigung der Ungulaten. PhD Dissertation, Universität Basel. 1895.

3. Dean MC, Reid DJ. Anterior tooth formation times in Australopithecus and Paranthropus. Twelfth International Symposium on Dental Morphology. University of Sheffield: Sheffield Academic Press Ltd.; 2001. pp. 135–149.

4. R Development Core Team. R: A Language and Environment for Statistical Computing [Internet]. Vienna, Austria: R Foundation for Statistical Computing; 2012. Available: http://www.R-project.org/

5. Wickham H. ggplot2: elegant graphics for data analysis [Internet]. New York: Springer Publishing Company, Incorporated; 2009. Available: http://had.co.nz/ggplot2/book

6. Lacruz RS. Enamel microstructure of the hominid KB 5223 from Kromdraai, South Africa. Am J Phys Anthropol. 2007;132: 175–182.

7. Beynon AD, Dean MC. Distinct dental development patterns in early fossil hominids. Nature. 1988;335: 509–514.

8. Bromage TG, Dean MC. Re-evaluation of the age at death of immature fossil hominids. Nature. 1985;317: 525–527. doi:10.1038/317525a0

9. Elhechmi I, Braga J, Dasgupta G, Gharbi T. Accelerated measurement of perikymata by an optical instrument. Biomed Opt Express. 2013;4: 2124–2137.

10. Elhechmi I. Instrumentation optique pour la mesure des périkymaties de la couronne dentaire. PhD Dissertation, Université de Franche-Comté. 2010.

11. Dean MC. Retrieving chronological age from dental remains of early fossil hominins to reconstruct human growth in the past. Philos Trans R Soc B Biol Sci. 2010;365: 3397–3410.

12. Mahoney EK, Rohanizadeh R, Ismail FSM, Kilpatrick NM, Swain MV. Mechanical properties and microstructure of hypomineralised enamel of permanent teeth. Focus Biomater Sci Aust. 2004;25: 5091–5100. doi:10.1016/j.biomaterials.2004.02.044

13. Boyde A. A review of problems of interpretation of the SEM image with special regard to methods of specimen preparation. Scanning Electron Microsc. 1971;1971: 1–8.

14. Boyde A. Quantitative photogrammetric analysis and qualitative stereoscopic analysis of SEM images. J Microsc. 1973;98: 452–471.

1. ***Report of the possibility of an imaging artifact resembling perikymata subdivisions when using PPC-SRµCT.***
   1. ***Original observations based on PPC-SRµCT data.***

In some regions, we noticed the existence of fine grooves between perikymata, which correspond to internal subdivisions of Retzius lines (Fig. 5) that could sometimes be misleading during long-period line counting. They occur locally in the middle of the traditionally defined structures, dividing perikymata and Retzius bands into two roughly equivalent halves.

These subdivisions of perikymata appear to be mostly a local phenomenon, as there are isochronous areas with and without such subdivisions (S7 Figure). A variability of this feature has also been observed on both the KB5223 LRI2 and LLI2 (S7 Figure, unequal subdivisions in the top and equal subdivisions at the bottom of the right inset), where the two halves are unequal in size, thus minimizing mistakes when counting. Nevertheless, in poorly preserved tooth surfaces that do not permit cross-checking counts at different positions, these subdivisions can lead to errors, especially when equal in size. Observers aware of this phenomenon can usually detect this issue. The subdivisions are also expressed, although in a fainter way, in the URI2 of MLD11-30 and locally on the distal edge of the LLI1 of SK62. The right canine of MLD11-30 shows both equal and unequal subdivisions, which become difficult to differentiate when the perikymata are highly packed as in the cervical area (Fig. 4 and S2 Movie, most visible on the last frames).

- 1. ***Long-period lines subdivisions in the literature.***

FitzGerald [1,2] reported observations of equal subdivisions of Retzius lines (termed “pseudo-striae” or “chevron lines”) from transmitted light microscopy. He noted that perikymata are not associated with these chevron lines [2] and therefore that “true” Retzius lines should be defined as those that meet the surface of the tooth and form perikymata [also see 3,4]. We also note that Ramirez-Rozzi [5] uses the term “chevron course” to describe a particular shape of Retzius line, which is different from what we report in the present study. Chevron lines as subdivisions of Retzius lines are also evident in Fig. 3 of Beynon *et al.* [6; Don Reid, pers. com.]. However, the Retzius line subdivisions observed in the current study do correspond to subdivisions of perikymata (Fig. 5) and appear to be different from the chevron lines that FitzGerald [1,2] illustrated and described. Moreover, we observe variation in the relative size of the subdivisions of Retzius lines and perikymata. S. Hillson has also noticed unequally-sized perikymata subdivisions by SEM observation of archeological teeth [7: fig. 4.5, and pers. com.]. This could however still be ambiguous when the subdivisions separate the perikymata into two equal halves (S7 Figure). Risnes [8] defined perikymata as continuous structures running around the circumference of the crown, in 3D correspondence with the Retzius lines, requiring observation of the tooth under multiple angles, as in S1 Figure. As already noticed by previous researchers, Retzius lines and perikymata should be observed at multiple observational scales and angles, and structures presenting inconsistent morphology and/or spacing within the enamel or on the tooth surface should be disregarded during quantification of long-period line or periodicity. These recommendations could be broadened to any incremental growth feature, including peri-radicular bands (Fig. 8) and increments in the dentine. For using these synchrotron-based techniques, we have however noticed that managing to obtain a clean and exploitable root surface is often tricky due to taphonomic damage of the root dentine or of a direct contact between the sedimentary matrix or the bone with the root surface (i.e. discontinuous and well-defined fringes of contrast cannot be segmented to yield the real physical root surface).

- 1. ***Main causes and interpretations.***

After suspicion of technological artifacts triggered during the revision process of this paper, we investigated whether these subdivisions could not have been at least partially induced by the X-ray imaging approaches.

By scrutinizing the virtual 2D sections (full resolution of the 5 µm datasets) of the KB5223 incisors at the locations where the strongest expression of the perikymata subdivision was observed, we realized that this corresponds to areas of demineralization. Indeed, we wish to recall that the KB5223 incisors have been subjected to strong taphonomical alteration (see the virtual histological slices in [9] showing a clear degradation of the inner tissues of the KB5223 teeth).

We focus on one zone of the labial KB5223 LLI2 crown illustrating an area where subdivisions have been observed (S8 Figure) and an area lacking those structures (S9 Figure). For both figures, the bottom orientation of the colored light source 2 was preferred to better reveal the topographical details of interest. Both illustrations use the same color-coding: green arrows for true perikymata/Retzius lines, and red arrows for perceived perikymata subdivisions.

On the 3D model of the OES (outer enamel surface) at high resolution (0.7 µm), it clearly appears that those subdivisions are lacking (both on S8 Figure-A and on S9 Figure-A: green frame). The demineralization is still visible in 2D as a large black spot below the OES (S8 Figure-B and see the inset from the yellow frame), but the phase contrast fringe defining the OES is still thick enough (S8 Figure-B: red double-arrows) not to create the depression seen and mistaken for a subdivision as visible on the 5 µm dataset (S8 Figure-C for the 3D and S8 Figure-D for the 2D). This type of demineralization of the enamel sub-surface is very similar to what is described in studies on alterations of enamel microstructure [10,11]. This explains the visibility of the prisms at the enamel surface in S8 Figure-A and S9 Figure-A (insets). The 0.7 µm dataset was voluntarily degraded^[[1]](#footnote-1)^ to simulate the aspect of the 5 µm scans (S8 Figure-E for the 3D and S8 Figure-F for the 2D), to rule out that these observations could be due to the acquisition itself. On the 2D virtual section generated from both the 5 µm and the degraded 0.7 µm datasets (S8 Figure-D and F), there is an apparent reduction of the thickness and maximum white level of the white fringe between enamel and air, visible as a lighter gray blob in between the two Retzius lines reaching the enamel surface (double red arrows). This is related to the partial volume effect [12 see Figs. 3&4, 13 see Fig. 2]: the real surface topography is not affected by the underlying demineralization. However, since the 3D rendering algorithms are based on the detection of gray levels, this local decrease in gray levels, caused by the partial volume effect on a thin structure, finally leads to a modification of the topography when the resolution is too low. This phenomenon is not a result of phase contrast, as it would also occur when acquiring data in absorption mode with either a conventional or a synchrotron source (provided that the resolution is high enough to image the perikymata). Because of the demineralization underneath the enamel surface, this latter would still appear at a darker gray level than the surrounding surface, and this would disturb the continuity of the surface when using any gray level-based rendering or segmentation process. Since the fringe in between two real perikymata (green arrows in S8 Figure) is weakened by a depression caused by the demineralization running parallel to the perikymata, this creates an artificial perikymata subdivision. S9 Figure shows that for the three datasets (0.7 µm, 5 µm and degraded 0.7 µm) in a zone lacking perikymata/Retzius lines subdivisions. There is no substantial demineralization of the enamel subsurface (see the 2D virtual slices in S9 Figure-B, D and F), guaranteeing a continuous white fringe of consistent thickness and a regular perikymata ridge (see S9 Figure-A, C and E). It is worth stressing that these areas of sub-surface demineralization on the KB5223 teeth do not randomly occur. They originated from actual subdivisions of the Retzius lines, that are visible on the virtual slices and that look similar to the structures described by FitzGerald [1,2]. In the latter case, their biological origin seems to be well established, even though it is still not really understood. These 2D structures are relatively common, but do not normally lead to any modification of the OES topography. On this respect PPC-SRμCT actually revealed artifactually-induced perikymata subdivisions, only because these areas of demineralization were extremely close to the surface (< 30 µm). Their aspect on the 3D renderings, with their apparent trajectory running parallel to the perikymata, is related to their biological origin. It has to be noticed that, from place to place, these spots of demineralization do reach the surface, and appear as localized subdivisions of the perikymata which are visible under a binocular microscope (S10 Figure). They would nonetheless not be mistaken for perikymata. We would like to stress that although this was confusing our counts at the beginning, the investigation of this phenomenon allowed us to trust the structures we were counting and finally reporting.

There are however places where an *unequal* subdivision of the perikymata occur and that are not accentuated by X-ray imaging, as seen on the binocular, SEM and PPC-SRµCT versions of S6 Figure. This split is nonetheless much more superficial than a real perikymata groove, and looks like a thin layer covering a small part of the perikymata ridge [7, Fig.4.5]. This does not systematically produce a visible subdivision when generating a 3D model with the 5 µm datasets (full resolution or binning 2). On S8 Figure (turquoise frame), this thin structure overlaying the perikymata is visible on the three datasets, but would not affect perikymata counts. S10 Figure illustrates the case of a stronger expression of these perikymata subdivisions visible on both PPC-SRµCT and stereomicroscopic images. In this situation, the demineralization of the enamel subsurface may reach the OES to create this non-artifactual structure.

- 1. ***Literature cited***

1. FitzGerald CM. Do enamel microstructures have regular time dependency? Conclusions from the literature and a large-scale study. J Hum Evol. 1998;35: 371–386.

2. FitzGerald CM. Tooth crown formation and the variation of enamel microstructural growth markers in modern humans. PhD Dissertation, University of Cambridge. 1995.

3. Smith TM. Incremental development of primate dental enamel. PhD Dissertation, Stony Brook University. 2004.

4. Dean MC. The dental developmental status of six East African juvenile fossil hominids. J Hum Evol. 1987;16: 197–213.

5. Ramirez Rozzi F. Can enamel microstructure be used to establish the presence of different species of Plio-Pleistocene hominids from Omo, Ethiopia? J Hum Evol. 1998;35: 543–576.

6. Beynon AD, Dean MC, Reid DJ. On thick and thin enamel in hominoids. Am J Phys Anthropol. 1991;86: 295–309.

7. Hillson S. Tooth development in human evolution and bioarchaeology. Cambridge University Press; 2014.

8. Risnes S. Growth tracks in dental enamel. J Hum Evol. 1998;35: 331–350.

9. Smith TM, Tafforeau P, Le Cabec A, Bonnin A, Houssaye A, Pouech J, et al. Dental Ontogeny in Pliocene and Early Pleistocene Hominins. PloS One. 2015;10: e0118118. doi:10.1371/journal.pone.0118118

10. Boyde A, Jones SJ, Reynolds PS. Quantitative and qualitative studies of enamel etching with acid and EDTA. Scanning Electron Microsc. 1978;2: 991–1002.

11. Li C, Risnes S. SEM observations of Retzius lines and prism cross-striations in human dental enamel after different acid etching regimes. Arch Oral Biol. 2004;49: 45–52. doi:10.1016/S0003-9969(03)00195-X

12. Vincken KL, Koster AE, Viergever MA. Probabilistic segmentation of partial volume voxels. Vol Image Process VIP93. 1994;15: 477–484. doi:10.1016/0167-8655(94)90139-2

13. Bouxsein ML, Boyd SK, Christiansen BA, Guldberg RE, Jepsen KJ, Müller R. Guidelines for assessment of bone microstructure in rodents using micro–computed tomography. J Bone Miner Res. 2010;25: 1468–1486. doi:10.1002/jbmr.141

1. ***Cuspal daily secretion rates (DSR) for StW151 and MLD2 LLC crown formation time (CFT) calculations.***
   1. ***Cuspal DSR from the literature.***

To our knowledge, only cuspal DSR for molars are available in the literature for fossil hominins. We have collected the DSR and equations (from canines and molars of modern humans and great apes, and from molars for fossil hominin) to calculate the cuspal formation time of our two teeth of interest. We would like to remind that those rates are often based on small sample sizes and on teeth belonging to different individuals (which is perfectly understandable provided the physical sections required in classical histology). We would like to stress that some of the published equations have been corrected after communication with the concerned authors, following editorial typos. This is clearly indicated in the results. To report a wider range increasing the probability to include the “real” value of our specimens, we also consider reporting cuspal DSR for great apes and recent modern humans. The different cuspal time obtained for both StW151 and MLD2 LLC are presented in the S3 Supporting Information.

- 1. ***Attempts of direct measurements of cuspal DSR.***

A direct measurement of cuspal DSR would be ideal on each specimen. Unfortunately, when the fossils were brought to the ESRF for scanning, for a matter of beam time and of not sensitive enough scanning protocols for successfully imaging the cuspal region, no high resolution (~0.7 µm; HR) scans could be performed along a transect in the cuspal enamel of any (anterior) teeth. Although this is not as accurate as an HR scan would be, we have attempted to measure the cuspal DSR in several canines on the full resolution versions of the 5 µm scans.

Cuspal DSR are conventionally measured by counting cross-striations on sagittal sections of the tooth of interest. When working on the PPC-SRµCT scans at a relatively low resolution (i.e., 5 µm for investigating the long-period lines), it appears nearly impossible to distinguish cuspal Retzius lines from accentuated stress lines, as the regularity of their pattern cannot be safely identified. It is furthermore well-known among dental histologists that counting cuspal Retzius lines on physical sections is very challenging if not impossible. Nevertheless, we noticed that using a thick transversal slice in the 5 µm PPC-SRµCT data enhanced the visibility of the long-period lines, especially by improving the assessments of their regularity in spacing (S11 Figure - C and E). Since a canine cusp tip can be understood as a series of interlocked cones, Retzius lines appear as concentric lines. In anterior teeth, there is a particular area at the very top of the lateral enamel before the strong curvature of the cusp tip, where all the Retzius lines appear to be locally straight and parallel to each other (S11 Figure – D). As PPC-SRμCT-based virtual histology is relying on thick slices perfectly oriented along the structures of interest [1], exploring a transversal slice centered in this specific region, and oriented perpendicularly to the Retzius lines, allows using highly thick slices (up to 300 μm). This does not result into any blurring of the superimposed structures. The incremental pattern becomes far more visible than on sagittal slices recorded at the same location. We traced those increments along a quarter of circle, we then used accentuated lines to match this count back to the sagittal section to identify the cuspal thickness from the last perikymata to the dentine horn tip, and even try to differentiate inner, middle and outer DSR.

This yielded agreeable results on the one hand, in MLD11-30 URC and STS2 ULC (both *Au. africanus*), and on the other hand in StW151 ULC (South African *Homo* or *Au. africanus*). The long-period growth lines were the best and the most reliably visible in those three teeth. Absolutely no microstructure could be distinguished in the MLD2 LLC which directly excluded this specimen for the measurement. Regarding StW151, the cuspal microstructure was most visible in the ULC, but since this is the same tooth class (as the studied LLC), we assume that the results will anyway be more relevant than any molar rates. We present the pictures used for the measurements in S11 Figure.

- - 1. ***STS2 ULC*** *(Au. africanus)*

Periodicity = 11 days [2]; counts on a 200 µm-thick transversal slab in the cuspal area near the standardized developmental section.

|  |  | | **Outer** | |  | |  | |  | | **Middle** | |  | | |  |  | | **Inner** | |  | |
| --- | --- | --- | --- | --- | --- | --- | --- | --- | --- | --- | --- | --- | --- | --- | --- | --- | --- | --- | --- | --- | --- | --- |
| (Observers) | A | B | | Average | |  | | A | | B | | Average | |  | A | | | B | | Average | |  |
| Retzius lines | 12 | 12 | | 12 | |  | | 16 | | 16 | | 16 | |  | 12 | | | 12 | | 12 | |  |
| Local cuspal thickness [µm] | 413 | 413 | | 413 | |  | | 478 | | 478 | | 478 | |  | 410 | | | 410 | | 410 | |  |
| **DSR [µm/d]** | 3.13 | 3.13 | | **3.13** | |  | | 2.72 | | 2.72 | | **2.72** | |  | 3.11 | | | 3.11 | | **3.11** | |  |

Total cuspal DSR average = **2.98** **µm/d.**

Total cuspal formation time = (12+16+12)*11=40*11=**440 days.**

Cuspal DSR rate used in Smith et al. [2] = 5.53 µm/d (corrected from [3]) with a 1050 µm- thick cuspal enamel in STS2 ULC => 190 days of cuspal formation time [2].

Applying the molar rate for *Au. africanus* seem to considerably underestimate the cuspal formation time.

- - 1. ***MLD11-30 URC*** *(Au. africanus)*

Periodicity = 6 days [2]; counts on a 200 µm-thick transversal slab in the cuspal area near the standardized developmental section.

|  |  | **Outer** |  |  |  | **Middle** |  |  |  | **Inner** |  |
| --- | --- | --- | --- | --- | --- | --- | --- | --- | --- | --- | --- |
| (Observers) | A | B | Average |  | A | B | Average |  | A | B | Average |
| Retzius lines | 17 | 17 | 17 |  | 20 | 20 | 20 |  | 17 | 19 | 18 |
| Local cuspal thickness [µm] | 511 | 514 | 512.5 |  | 480 | 493 | 486.5 |  | 452 | 458 | 455 |
| **DSR [µm/d]** | 5.01 | 5.04 | **5.02** |  | 4.00 | 4.11 | **4.05** |  | 4.43 | 4.02 | **4.22** |

Total cuspal DSR average = **4.43** **µm/d.**

Total cuspal formation time - Obs. A: (17+20+17)*6=54*6= **324 days.**

- Obs. B: (17+20+19)*6=56*6= **336 days.**

Cuspal DSR rate used in Smith et al. [2] = 5.53 µm/d (corrected from [3]) with a 1395 µm- thick cuspal enamel in MLD11-30 URC => 252 days of cuspal formation time [2].

- - 1. ***StW151 ULC*** *(Au. africanus* or South African *Homo)*

Periodicity = 8 days [2]; counts on a 300 µm-thick transversal slab in the cuspal area near the standardized developmental section. Projection of accentuated lines and last perikymata on a 150 µm-thick sagittal slice.

|  |  | **Outer** |  |  |  | **Middle** |  |  |  | **Inner** |  |
| --- | --- | --- | --- | --- | --- | --- | --- | --- | --- | --- | --- |
| (Observers) | A | B | Average |  | A | B | Average |  | A | B | Average |
| Retzius lines | 9 | 9 | 9 |  | 13 | 13 | 13 |  | 11 | 12 | 11.5 |
| Local cuspal thickness [µm] | 245 | 245 | 245 |  | 340 | 340 | 340 |  | 298 | 298 | 2.98 |
| **DSR [µm/d]** | 3.40 | 3.40 | **3.40** |  | 3.27 | 3.27 | **3.27** |  | 3.39 | 3.10 | **3.25** |

Total cuspal DSR average = **3.31** **µm/d.**

Total cuspal formation time - Obs. A: (9+13+11)*8=33*8= **264 days.**

- Obs. B: (9+13+12)*8=34*8= **272 days.**

Cuspal DSR rate used in Smith et al. [2]: - *Au. africanus* DSR= 5.53 µm/d (corrected from [3]) with a 1175 µm- thick cuspal enamel in StW151 ULC => 212 days of cuspal formation time [2].

- South African early *Homo* DSR= 4.44 µm/d (DSR average of *H. habilis* and *H. rudolfensis* from [3]) with a 1175 µm-thick cuspal enamel in StW151 ULC => 212 days of cuspal formation time [2].

- 1. ***Resulting overall ranges (see S3 Supporting Information).***
     1. ***MLD2 LLC***

Our mean measured CuDSR is 3.57 µm/d, yielding a CFT of 5.03 ± 0.25 years for the MLD2 LLC. We report the following ranges:

|  |  | CFT | Sources for CuDSR |
| --- | --- | --- | --- |
| For anterior | Min. | 4.86 - 0.25 = 4.61 years | *Pan* upper canine at 4.40 µm/d [4]. |
| teeth |  |  | and MLD11-30 URC at 4.43 µm/d |
|  |  |  | [This study] |
|  | Max. | 5.16 + 0.25 = 5.41 years | *Homo sapiens* canine at 3.00 µm/d [5] |
| Including | Min. | 4.69 - 0.25 = 4.44 years | DNH35 South African *Homo* M1 at 6.06 |
| molars |  |  | µm/d [2] |

- - 1. ***StW151 LLC***

Our mean measured CuDSR is 3.57 µm/d, yielding a CFT of 4.83 ± 0.15 years for the StW151 LLC. We remind that this tooth is not crown complete, involving that the time provided here is smaller than the real CFT. We report the following ranges:

|  |  | CFT | Sources for CuDSR |
| --- | --- | --- | --- |
| For anterior | Min. | 4.61 - 0.15 = 4.46 years | *Pan* upper canine at 4.40 µm/d [4]. |
| teeth |  |  | and MLD11-30 URC at 4.43 µm/d |
|  |  |  | [This study] |
|  | Max. | 4.92 + 0.15 = 5.07 years | *Homo* sapiens canine at 3.18 µm/d [5] |
| Including | Min. | 4.39 - 0.15 = 4.24 years | DNH35 South African *Homo* M1 at 6.06 |
| molars |  |  | µm/d [2] |

- 1. ***Concluding remarks***

Despite the fact that this approach based on transversal slices is not as precise as a direct observation of cross-striations on high resolution data, and that it would require a more extensive validation, it tends to indicate that published molar CuDSR would be consistently higher than canine CuDSR, leading most probably to an underestimation of the cuspal formation time, and thereby of the total crown formation time. Interestingly, our measured CuDSRs fall within (or very close) to those measured on canines of modern humans and chimpanzees (average rates of 3 μm/day for *Homo sapiens* [5] and 4.4 μm/day for *Pan troglodytes* [4], see S3 Supporting Information).

- 1. ***Cited literature***

1. Tafforeau P, Bentaleb I, Jaeger J-J, Martin C. Nature of laminations and mineralization in rhinoceros enamel using histology and X-ray synchrotron microtomography: potential implications for palaeoenvironmental isotopic studies. Palaeogeogr Palaeoclimatol Palaeoecol. 2007;246: 206–227.

2. Smith TM, Tafforeau P, Le Cabec A, Bonnin A, Houssaye A, Pouech J, et al. Dental Ontogeny in Pliocene and Early Pleistocene Hominins. PloS One. 2015;10: e0118118. doi:10.1371/journal.pone.0118118

3. Lacruz RS, Dean MC, Ramirez‐Rozzi F, Bromage TG. Megadontia, striae periodicity and patterns of enamel secretion in Plio-Pleistocene fossil hominins. J Anat. 2008;213: 148–158.

4. Reid DJ, Schwartz GT, Dean C, Chandrasekera MS. A histological reconstruction of dental development in the common chimpanzee, *Pan troglodytes*. J Hum Evol. 1998;35: 427–448. doi:10.1006/jhev.1998.0248

5. Schwartz GT, Dean C. Ontogeny of canine dimorphism in extant hominoids. Am J Phys Anthropol. 2001;115: 269–283. doi:10.1002/ajpa.1081

1. This voluntarily degraded dataset was obtained by applying a binning (factor 4 applied in Matlab) on the full resolution 0.7 µm dataset (yielding a pixel size of 2.8 µm) and then three iterations of a Gaussian blur filter (5 pixels) in VGStudioMAX2.2. This processing has generated artifacts visible on the surface of the 3D model (rings on S8 and S9 Figures-E) but this does not impact the purpose of the demonstration. [↑](#footnote-ref-1)
