# Supplementary material for: Accessing Developmental Information of Fossil Hominin Teeth Using New Synchrotron Microtomography-Based Visualization Techniques of Dental Surfaces and Interfaces
Source: PLoS One. 2015 Apr 22;10(4):e0123019. doi: 10.1371/journal.pone.0123019 (PMC4406681; doi:10.1371/journal.pone.0123019)
Supplement: S1 Table — File name: S1_Table.docx. (DOCX) [file pone.0123019.s020.docx]

**S1 Table. Sample of fossil hominin teeth included in this study.**

| **Specimen** | **Curating institution** | **Site name and its** | **Dating** | **Taxon** | **Tooth type** |
| --- | --- | --- | --- | --- | --- |
|  |  | **location** |  |  |  |
| KNM-KP | National Museums of | Kanapoi, Kenya | 4.17 ± 0.03 – 4.07± 0.03 Ma [1] | *Australopithecus* | LRC, LRM1 |
| 34725 | Kenya |  |  | *anamensis* |  |
| MLD2 | University of the | Makapansgat, South | ~3 Ma [2,3] | *Australopithecus* | LLM1, LLM2, |
|  | Witwatersrand | Africa | 2.4–2.8 Ma [4] | *africanus* | LLC |
| MLD11-30 | University of the | Makapansgat, South | ~3 Ma [2,3] | *Australopithecus* | URI2, URC |
|  | Witwatersrand | Africa | 2.4–2.8 Ma [4] | *africanus* |  |
| KB5223 | DITSONG Museums | Member 3, Kromdraai | ~1.9 Ma [5] | *Paranthropus* | LLI1, LLI2 |
|  | of South Africa | B, South Africa |  | *robustus* |  |
| StW151 | University of the | Member 4, | 2.8 – 2.6 Ma [6] | *Au. africanus?* | ULC, ULM1, |
|  | Witwatersrand | Sterkfontein, South |  | *Early Homo?* | LLC |
|  |  | Africa |  |  |  |
| SK62 | DITSONG Museums | Member 1, | 1.8 – 1.6 Ma [7,8] | *Paranthropus* | LLI1 |
|  | of South Africa | Swartkrans, South |  | *robustus* |  |
|  |  | Africa |  |  |  |
| STS2 | DITSONG Museums | Member 4, | 1.5 – 2.5 Ma [9] | *Australopithecus* | ULC |
|  | of South Africa | Sterkfontein, South |  | *africanus* |  |
|  |  | Africa |  |  |  |
| STS24 | DITSONG Museums | Member 4, | 1.5 – 2.5 Ma [9] | *Australopithecus* | LRI1, URI1, |
|  | of South Africa | Sterkfontein, South |  | *africanus* | ULI2 |
|  |  | Africa |  |  |  |

For the tooth type: the first letter ‘L’ or ‘U’ stands for ‘lower’ (mandibular) and ‘upper’ (maxillary), respectively. The second letter ‘L’ or ‘R’ indicates the side (‘left’ or ‘right’ respectively). The last part of the labeling is as: I1= central incisor; I2=lateral incisor; C= canine; M1= first molar; M2 = second molar.

**References**

1. Leakey MG, Feibel CS, McDougall I, Ward C, Walker A (1998) New specimens and confirmation of an early age for *Australopithecus anamensis*. Nature 393: 62–66.

2. Cadman A, Rayner RJ (1989) Climatic change and the appearance of *Australopithecus africanus* in the Makapansgat sediments. J Hum Evol 18: 107–113. doi:10.1016/0047-2484(89)90065-1.

3. Rayner RJ, Moon BP, Masters JC (1993) The Makapansgat australopithecine environment. J Hum Evol 24: 219–231.

4. De Ruiter D, Churchill S, Berger L (2013) *Australopithecus sediba* from Malapa, South Africa. In: Reed KE, Fleagle JG, Leakey RE, editors. The Paleobiology of *Australopithecus*. Vertebrate Paleobiology and Paleoanthropology. Springer Netherlands. pp. 147–160. Available: http://dx.doi.org/10.1007/978-94-007-5919-0_9.

5. Thackeray JF, Kirschvink JL, Raub TD (2002) Palaeomagnetic analyses of calcified deposits from the Plio-Pleistocene hominid site of Kromdraai, South Africa. South Afr J Sci 98: 537–540.

6. Moggi-Cecchi J, Tobias PV, Beynon AD (1998) The mixed dentition and associated skull fragments of a juvenile fossil hominid from Sterkfontein, South Africa. Am J Phys Anthropol 106: 425–465.

7. Pickering R, Kramers JD, Hancox PJ, de Ruiter DJ, Woodhead JD (2011) Contemporary flowstone development links early hominin bearing cave deposits in South Africa. Earth Planet Sci Lett 306: 23–32. doi:10.1016/j.epsl.2011.03.019.

8. Cofran ZD (2012) Mandibular Growth in *Australopithecus robustus* [PhD Dissertation]. Wellesley College.

9. Berger LR, Lacruz R, De Ruiter DJ (2002) Revised age estimates of *Australopithecus*-bearing deposits at Sterkfontein, South Africa. Am J Phys Anthropol 119: 192–197. doi:10.1002/ajpa.10156.
