# Supplementary material for: Accessing Developmental Information of Fossil Hominin Teeth Using New Synchrotron Microtomography-Based Visualization Techniques of Dental Surfaces and Interfaces
Source: PLoS One. 2015 Apr 22;10(4):e0123019. doi: 10.1371/journal.pone.0123019 (PMC4406681; doi:10.1371/journal.pone.0123019)
Supplement: S2 Table — File name: S2_Table.docx. (DOCX) [file pone.0123019.s021.docx]

**S2 Table. Scanning parameters for the acquisition of phase contrast synchrotron micro-CT data.**

| Specimen | MLD11-30 | MLD2 | MLD2 | KB5223 | KNM-KP34725 | STS2 |
| --- | --- | --- | --- | --- | --- | --- |
| Tooth type | URI2, URC | LLM1, LLM2 | LLC | LLI1, LLI2 | LRC, LRM1 | ULC |
| Average Energy | 67 keV | 132 keV | 167 keV | 91 keV | 91 keV | 67 keV |
| Wiggler W150 gap | 62 mm | 37 mm | 28 mm | 55 mm | 55 mm | 62 mm |
| Filters | 2 mm Al, 0.25 mm | 2 mm Al, 8 mm Cu | 2 mm Al, 8 mm | 2 mm Al, 3 mm Cu | 2 mm Al, 3 mm Cu | 2 mm Al, 0.25 mm |
|  | Cu, 0.25 mm W |  | Cu, 0.25 mm W |  |  | Cu, 0.25 mm W |
| Voxel size | 4.96 µm | 4.96 µm | 4.96 µm | 4.96 µm | 4.96 µm | 4.96 µm |
|  |  |  |  |  |  |  |
|  |  |  |  |  |  |  |
| Specimen | SK62 | STS24 | STS24 | StW151 | StW151 |  |
| Tooth type | LLI1 | LRI1, ULI2 | URI1 | ULC, ULM1 | LLC |  |
| Average Energy | 110 keV | 67 keV | 67 keV | 91 keV | 51 keV |  |
| Wiggler W150 gap | 55 mm | 62 mm | 62 mm | 55 mm | - |  |
| Filters | 2 mm Al, 3 mm Cu | 2 mm Al, 0.25 mm | 2 mm Al, 0.25 mm | 2 mm Al, 3 mm Cu | - |  |
|  |  | Cu, 0.25 mm W | Cu, 0.25 mm W |  |  |  |
| Voxel size | 4.96 µm | 4.96 µm | 4.96 µm | 4.96 µm | 4.95 µm |  |
|  |  |  |  |  |  |  |
|  |  |  |  |  |  |  |

Teeth were scanned on the ID 19 beamline, with 4 meters of propagation distance using different optimized polychromatic beam configurations (except for StW151 LLC where a monochromatic configuration was used). The detector was composed of a 200 µm LuAg:Ce (Cerium-doped Lutetium Aluminum Garnet) scintillator, coupled via visible-light objectives to a FReLoN 2K (Fast Readout Low Noise, type: F_A7899) camera.

For the tooth type: the first letter ‘L’ or ‘U’ stands for ‘lower’ (mandibular) and ‘upper’ (maxillary), respectively. The second letter ‘L’ or ‘R’ indicates the side (‘left’ or ‘right’ respectively). The last part of the labeling is as: I1= central incisor; I2=lateral incisor; C= canine; M1= first molar; M2 = second molar.
