# Supplementary material for: Accessing Developmental Information of Fossil Hominin Teeth Using New Synchrotron Microtomography-Based Visualization Techniques of Dental Surfaces and Interfaces
Source: PLoS One. 2015 Apr 22;10(4):e0123019. doi: 10.1371/journal.pone.0123019 (PMC4406681; doi:10.1371/journal.pone.0123019)
Supplement: S3 Table — File name: S3_Table.docx. (DOCX) [file pone.0123019.s022.docx]

**S3 Table.** **Inter-observer agreement study for all counting sessions (mean ± standard deviation).**

| **Specimen** | **Tooth type** | **Observer 1** | **Observer 2** | **Observer 3** | **Published data^1^** |
| --- | --- | --- | --- | --- | --- |
|  |  | **(4 counts)** | **(3 counts)** | **(4 counts)** |  |
| KB5223 | LRI2 | 88.5 ± 2.9 | 86.7 ± 1.7 | 83.8 ± 1.0 | 86 [1] |
|  |  |  |  |  | 86^2^ [2] |
|  |  |  |  |  | 97 [3] |
| SK62 | LLI1 | 71.8 ± 4.6 | 74.7 ± 1.5 | 70.0 ± 1.4 | 68 [1] |
|  |  |  |  |  | 64 [4] |
| MLD11-30 | URC | 122.5 ± 10.2 | 123.7 ± 0.6 | 111.8 ± 6.6 | 132 [1] |
| MLD11-30 | URI2 | 120.8 ± 3.8 | 129 ± 9.2 | 123.3 ± 9.4 | 119 [1] |
| STS24 | LRI1 | 110.8 ± 6.7 | 109.7 ± 7.1 | 118.5 ± 11.7 | 121 [1] |
| STS24 | URI1 | 148.8 ± 4.6 | 154 ± 7.8 | 147.3 ± 6.9 | 138 [1] |
|  |  |  |  |  | 135 [5] |
|  |  |  |  |  | 135 [4] |
| STS24 | ULI2 | 111.3 ± 6.6. | 109.3 ± 3.8 | 106.5 ± 5.4 | 107 [1] |
| MLD2 | LLC | 226.0 ± 10.1 | 218.0 ± 5.3 | 214.8 ± 18.3 | Tooth still in crypt. |
|  |  |  |  |  | No count ever published. |
| StW151 | LLC | 179.3 ± 3.9 | 171 ± 0 | 171.8 ± 9.6 | 121 ± 5 [6] |

Inter**-**observer agreement study on perikymata counting of seven Plio-Pleistocene hominin anterior teeth, performed on the 3D renderings from PPC-SRµCT data described in this study, and comparison with data from the literature.

^1^ Confirmation of the side used for the dental casts stems from Dean (pers. com.) for the various counts published by Dean and colleagues. There is however an ambiguity for the STS24 LRI1, for which they might have counted on the cast of the left tooth.

^2^ The KB5223 LRI2 was studied in Lacruz (2007) [2, see Fig. 5] although it is referenced as a mandibular I1 in the text (typo, Lacruz, pers. com.).

**References**

1. Dean MC, Reid DJ (2001) Anterior tooth formation times in *Australopithecus* and *Paranthropus*. Twelfth International Symposium on Dental Morphology. University of Sheffield: Sheffield Academic Press Ltd. pp. 135–149.

2. Lacruz RS (2007) Enamel microstructure of the hominid KB 5223 from Kromdraai, South Africa. Am J Phys Anthropol 132: 175–182.

3. Elhechmi I, Braga J, Dasgupta G, Gharbi T (2013) Accelerated measurement of perikymata by an optical instrument. Biomed Opt Express 4: 2124–2137.

4. Beynon AD, Dean MC (1988) Distinct dental development patterns in early fossil hominids. Nature 335: 509–514.

5. Bromage TG, Dean MC (1985) Re-evaluation of the age at death of immature fossil hominids. Nature 317: 525–527. doi:10.1038/317525a0.

6. Moggi-Cecchi J, Tobias PV, Beynon AD (1998) The mixed dentition and associated skull fragments of a juvenile fossil hominid from Sterkfontein, South Africa. Am J Phys Anthropol 106: 425–465.
